# Sex-linked deubiquitinase establishes uniparental transmission of chloroplast DNA

**DOI:** 10.1038/s41467-022-28807-6

**Published:** 2022-03-03

**Authors:** Sunjoo Joo, Thamali Kariyawasam, Minjae Kim, EonSeon Jin, Ursula Goodenough, Jae-Hyeok Lee

**Affiliations:** 1 Department of Botany, University of British Columbia, 6270 University Blvd, Vancouver, BC V6T 1Z4 Canada; 2 Department of Life Sciences, Hanyang University, Seoul, 133-791 Republic of Korea; 3 Department of Biology, Washington University in St. Louis, 1 Brookings Dr, St. Louis, MO 63110 USA

**Keywords:** Chloroplasts, Development, Plant reproduction

## Abstract

Most sexual organisms inherit organelles from one parent, commonly by excluding organelles from the smaller gametes. However, post-mating elimination of organelles derived from one gamete ensures uniparental inheritance, where the underlying mechanisms to distinguish organelles by their origin remain obscure. Mating in *Chlamydomonas reinhardtii* combines isomorphic *plus* and *minus* gametes, but chloroplast DNA from *minus* gametes is selectively degraded in zygotes. Here, we identify *OTU2p* (*otubain protein 2*), encoded in the *plus* mating-type locus *MT*+, as the protector of *plus* chloroplast. Otu2p is an otubain-like deubiquitinase, which prevents proteasome-mediated degradation of the preprotein translocase of the outer chloroplast membrane (TOC) during gametogenesis. Using *OTU2p*-knockouts and proteasome inhibitor treatment, we successfully redirect selective DNA degradation in chloroplasts with reduced TOC levels regardless of mating type, demonstrating that *plus*-specific Otu2p establishes uniparental chloroplast DNA inheritance. Our work documents that a sex-linked organelle quality control mechanism drives the uniparental organelle inheritance without dimorphic gametes.

## Abstract (web summary)

Most sexual organisms ensure that organelles are inherited from a single parent. Here, the authors describe OTU2p, a *Chlamydomonas* deubiquitinase that drives uniparental organelle inheritance without gametic dimorphism by preventing proteasome-mediated degradation exclusively in gametes of the *plus* mating type.

## Introduction

Sexual mating brings together two gametes, and meiotic products receive biparental nuclear genomes. Organellar genomes, by contrast, generally display maternal inheritance, commonly driven by a large size difference between egg and sperm cells. During animal fertilization, a small number of sperm mitochondria typically enter the egg, but these are rapidly eliminated^1,2^. The occasional paternal transmission of mitochondria has been linked to several human disorders^3,4^, implying the critical importance of strict maternal mitochondrial inheritance. Theoretical studies suggest that uniparental inheritance has evolved to mitigate the higher mutation rate observed in sperm mitochondria and to prevent potentially harmful interactions between heterologous organelles, including the spread of intracellular parasites and the disruption of coadaptation between the nuclear and organellar genomes^5,6^. Consistent with its proposed evolutionary advantages, uniparental organelle inheritance is ubiquitously observed in sexual organisms regardless of their gamete size^7,8^.

Inheritance of chloroplast DNA (cpDNA) has been extensively studied in the unicellular green alga *Chlamydomonas reinhardtii*, whose sexual mating combines isomorphic *plus* and *minus* gametes and yields a zygote that contains two chloroplasts, one from each gamete. However, the cpDNA from the *plus* parent (*plus* cpDNA) is selectively transmitted to meiotic progeny while *minus* cpDNA is largely absent^9,10^, indicating that mating type dictates which organellar chromosomes are to be inherited or excluded from progeny.

Biochemical and microscopic analysis of differentially labeled *plus* and *minus* cpDNA has shown that cpDNA in the *minus* chloroplast is selectively degraded within 1–2 h after gametic fusion^9,11^. Similar studies in diverse organisms also report selective destruction of organelles or organellar DNA during sexual mating^7,12^, suggesting self-clearance as a primary mechanism driving uniparental organelle inheritance apart from differential gamete sizes.

The molecular mechanisms by which mating types determine which organelles or organellar genomes to eliminate are, however, largely unknown. Furthermore, theoretical models predict that such self-destructive alleles are unlikely to become fixed due to their genetic conflict with the fittest cytotype, and their persistence has remained puzzling^13^. Therefore, understanding the mechanisms that distinguish and selectively degrade organelles by their parental origin will elucidate how sexual eukaryotes evolved uniparental organellar inheritance without incurring genetic conflicts.

In this study, we investigated how mating types control cpDNA inheritance in *C. reinhardtii* and identified a deubiquitinase, Otu2p, encoded in the *plus* mating-type locus, *MT*+, as a molecular determinant of selective cpDNA degradation. Our results provide a mechanistic model that explains the adaptive evolution of uniparental cpDNA transmission in *C. reinhardtii* and points to ancestral coevolution of organelle quality control and uniparental inheritance.

## Results

### Protection of cpDNA explains *MT*+-dominance in cpDNA inheritance

**Fig. 1:**
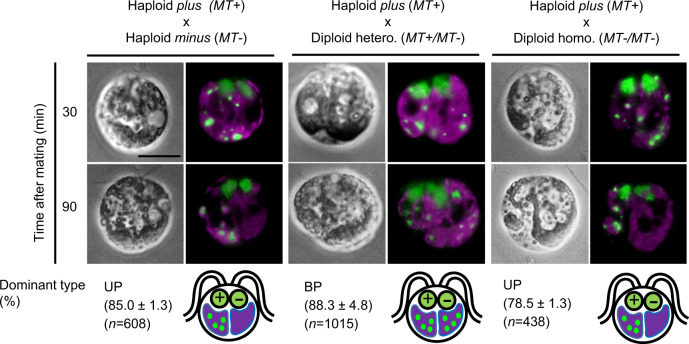
Chloroplast DNA from gametes harboring *MT*+ is protected from degradation in zygotes. Differential interference contrast and overlaid fluorescent images of early-stage zygotes were taken at 30 and 90 min after mating. Cells were stained with SybrG (green) for visualizing cpDNA, organized into nucleoids via punctate staining within chlorophyll autofluorescence (magenta), and nuclear DNA within two nuclei (see below diagrams for their locations). Complete cpDNA degradation is indicated by no green signals within one chloroplast. Bar = 5 μm. The below diagrams illustrate the early-stage zygote cells with two nuclei (green) and two chloroplasts (violet) with nucleoids (green dots). The percentage of the dominant cpDNA degradation pattern for each mating combination was calculated from three biological replicates (mean ± s.d.) “*n*” indicates the total number of zygotes examined.

Earlier genetic studies have documented that the *minus* mating-type *MT*− is dominant to *plus MT*+ in gametic differentiation: *MT*+/*MT−* and *MT−*/*MT−* diploids both differentiate as *minus* gametes. By contrast, *MT*+ is dominant in transmitting cpDNA to the progeny^14^ (Supplementary Fig. 1), indicating that *MT*+ either controls selective degradation directly or affects other pathways that bias cpDNA inheritance. To investigate the *MT*+-driven mechanism, we examined zygotes 2–3 h after mating by live-cell imaging, where the ~100 copies of cpDNA per chloroplast localize within 5–10 punctate clusters called nucleoids. In crosses between haploid *plus* and *minus* gametes, the punctate DNA stains in one chloroplast disappeared in >80% of the zygotes—the hallmark of uniparental cpDNA inheritance (UP; Fig. 1), while equivalent DNA staining between the two chloroplasts persisted in the remaining <20%. By contrast, in crosses between haploid *plus* gametes and *MT*+/*MT*− diploid *minus* gametes, >85% of the zygotes showed no signs of cpDNA degradation, while the remaining <15% showed disappearance of cpDNA staining from one chloroplast, the hallmark of biparental cpDNA inheritance (BP; Fig. 1). In control crosses between haploid *plus* gametes and *MT−*/*MT−* diploid gametes, the biparental zygote rate remained below 20%, comparable to the wild-type haploid crosses, documenting that the presence of *MT*+ in the *MT*+/*MT−* diploid, and not doubled ploidy, was responsible for the BP outcome. These observations and those in prior publications^11,14^ show that *MT*+ confers “protection” on cpDNA, sheltering it from degradation in the zygote, and hence is dominant to *MT−* which confers no such protection.

### *MT*+-encoded *OTU2p* protects chloroplast DNA from degradation

**Fig. 2:**
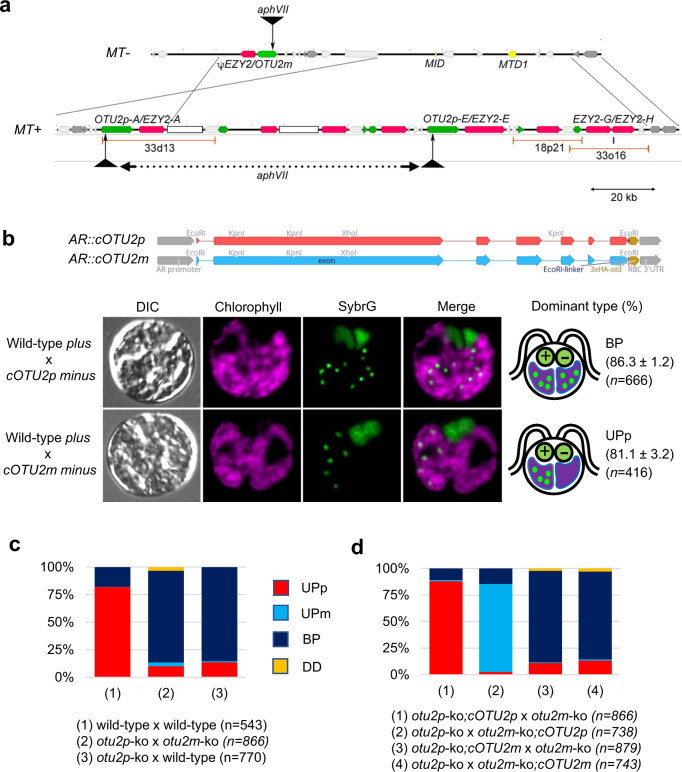
*MT*+ -linked *OTU2p* establishes the uniparental inheritance of cpDNA. **a** R-domain map of the mating-type loci *MT−* and *MT*+, focused on the 16-kb repeats unique to *MT*+. In the 16-kb repeats, *OTU2* copies (green) and *EZY2* copies (magenta) are alternating. BAC-plasmid clones used in this study are shown as red bars. The integration sites of *aphVII* delivered by CRISPR-Cas9 resulted in a ~80-kb deletion in *MT*+ (black triangles). **b**–**d** The average percentages of uniparental for *plus* (UPp), uniparental for *minus* (UPm), biparental (BP), and double degradation without cpDNA staining (DD) were calculated from three biological replicates. Chloroplasts from *plus* and *minus* gametes were distinguished by overlapping parent-specific mitochondrial staining of UPp vs. UPm (See Fig. 5c). “*n*” indicates the total number of zygotes examined. Stacked bar graphs show four cpDNA degradation patterns, whose detailed values are provided in Table S3. **b** Ectopic *cOTU2p* expression protects *minus* cpDNA from degradation. The above diagrams show plasmid maps of *AR::cOTU2p* and *AR::cOTU2m*. Differential interference contrast and overlaid fluorescent images of SybrG (green)-stained zygotes with chlorophyll autofluorescence (magenta) were taken at 90 min after mating. Bar = 5 μm. The percentage of the dominant cpDNA degradation pattern for each mating combination was calculated from three biological replicates (mean ± s.d.) “*n*” indicates the total number of zygotes examined. **c** Zygotes lacking OTU2p show biparental cpDNA inheritance. **d** Differential expression of *cOTU2p* between two gametes drives selective cpDNA degradation patterns in *otu2*-ko zygotes.

The *MT*+ and *MT*− loci occupy the same positions on linkage-group VI but encode distinctive gene sequences^15,16^. To understand how *MT*+ protects cpDNA from zygotic degradation, we focused on a 155-kb segment in *MT*+, divergent from its counterpart in *MT−*, which encodes at least two genes: *OTU2* (*otubain protein 2*) and *EZY2* (*early zygotic locus 2*), alternating in 16-kb repeats (Fig. 2a and Supplementary Fig. 2)^15,16^. The genetic dominance of the “protector” function of *MT*+ predicts that the protector would result in biparental cpDNA inheritance when introduced to *minus* gametes. Three bacterial artificial chromosomes (BAC) clones that harbor portions of the 155-kb segment were isolated and transformed into *minus* cells (Supplementary Fig. 3). With the 33d13 clone carrying full-length copies of *OTU2* and *EZY2*, five out of 13 transformants significantly increased the inheritance of a *minus* cpDNA marker for erythromycin resistance up to 28% of the progeny in crosses with *plus* gametes. In contrast, the 18p21 clone containing truncated copies of *OTU2* and the 33o16 clone containing two copies of *EZY2* had no effect (Supplementary Table 1). These results suggested that either *OTU2* is the *MT* + –linked “protector” of cpDNA or the combined action of *OTU2* and *EZY2* generates cpDNA protection.

The *MT*+ locus contains at least two nearly identical copies of *OTU2p*, whereas *MT−* includes a single copy called *OTU2m*^16^ (Fig. 2a and Supplementary Fig. 4). The protein sequences encoded by *OTU2p* (Otu2p) and *OTU2m* (Otu2m) differ at 39 of 1179 aligned amino acid (aa) residues (Supplementary Fig. 5). To test the role of Otu2p as the *MT*+ protector, a construct driving constitutive expression of the coding segment of *OTU2p* (*cOTU2p*) was introduced into *minus* cells. In six of the 15 *minus* strains expressing *cOTU2p*, the transmission of *minus* cpDNA marker for spectinomycin resistance significantly increased by three- to five-fold (67–97% of the progeny; Supplementary Table 2). Consistent with this transmission pattern, up to 86% of zygotes from the crosses of the resultant transgenic *minus* gametes retained cpDNA from both parents for up to 3.5 h after mating, as indicated by comparable staining of nucleoids in two chloroplasts (Fig. 2b). By contrast, the coding sequence of *OTU2m* (*cOTU2m*), when ectopically expressed in *minus* cells, did not significantly change the wild-type cpDNA inheritance pattern nor *minus*-selective cpDNA degradation in the resulting zygotes (Supplementary Table 2 and Fig. 2b). These results indicate that Otu2p can serve as a dominant protector in gametes with an *MT−* background, whereas Otu2m cannot perform this function.

The dominant role of Otu2p in protecting cpDNA predicted that *plus* gametes lacking Otu2p would leave *plus* chloroplasts unprotected, and all cpDNA would be degraded in the zygote, a lethal outcome. To examine the consequences of *OTU2* deletion, we generated *otu2* mutant strains (*otu2p*-ko and *otu2m*-ko) using CRISPR/Cas9-mediated targeted-insertion^17^ (Supplementary Fig. 6). Contrary to our prediction, nonselective cpDNA degradation was not evident in *otu2p*-ko x *otu2m*-ko zygotes (hereafter otu2pm-ko) nor *otu2p*-ko x wild-type *minus* zygotes (hereafter otu2p-ko). Instead, ~85% of zygotes lacking Otu2p displayed comparable cpDNA staining between two chloroplasts (Fig. 2c).

Comparable cpDNA staining between two chloroplasts in zygotes might be observed if cpDNA destruction is nonselective but is limited in scope. To assess whether or not cpDNA degradation occurred in the absence of Otu2p, we compared the total cpDNA quantity in wild-type and otu2pm-ko zygotes. Between 0.5 and 2 h after mating, the cpDNA amount was reduced by up to 50% in wild-type zygotes, expected if the *minus* cpDNA gets entirely degraded. In contrast, only a 5–10% reduction was observed in otu2pm-ko zygotes in the same period after mating (Supplementary Fig. 7), indicating that the zygote withheld cpDNA degradation when Otu2p was absent from both gametes.

We next tested whether *OTU2* transgene expression complements the defects observed in *otu2* knock-out strains. When the *cOTU2p* transgene was introduced into either *otu2p*-ko or *otu2m*-ko mutant, selective degradation resumed in >80% of the resulting zygotes, comparable to wild-type zygotes (Fig. 2d and Supplementary Table 3). Furthermore, in these UP zygotes, the cpDNA of the *cOTU2p*-expressing gametes was protected, while the cpDNA of gametes lacking *cOTU2p* expression was degraded. In contrast, *cOTU2m* expression did not restore selective degradation of cpDNA, indicating that differential *OTU2p* expression is sufficient to establish selective cpDNA degradation regardless of *OTU2m* expression.

### Otu2p differentiates TOC between *plus* and *minus* chloroplasts

**Fig. 3:**
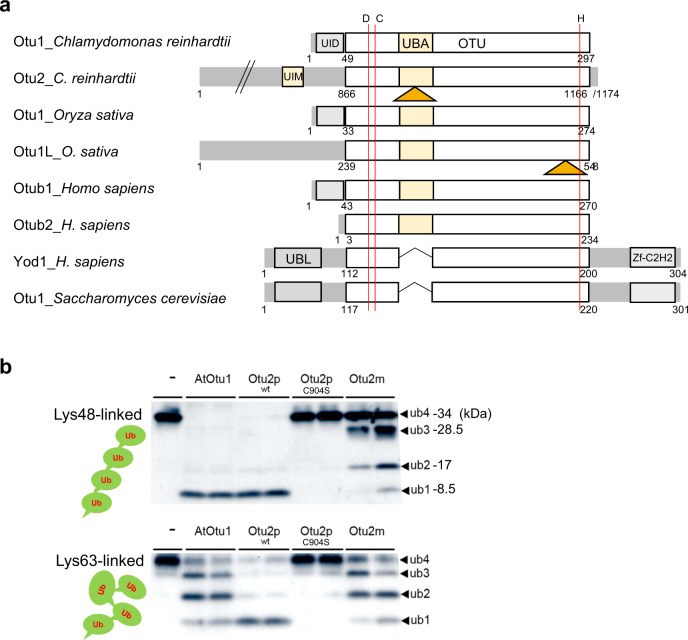
The deubiquitinase Otu2p protects TOCs from degradation during gametogenesis. **a** Schematic domain structure of OTUB (protease C65) family deubiquitinases (DUBs). OTUB family members are distinguished by a UBA (ubiquitin-associated) domain in the catalytic OTU domain and lack N-terminal UBL (UBX-like) and C-terminal zf-C2H2 domains conserved among OTUD proteins. *OTU2* encodes an 1166 or 1174 amino acid (aa) protein with a C-terminal OTU domain. Otu2 homologs include a unique loop (orange triangle, 55 aa-long in *C. reinhardtii*) within the UBA domain. The predicted catalytic triad (vertical red lines; D901, C904, and H1151 in Otu2_*C. reinhardtii*) is conserved in OTUB and OTUD. Divergent OTUB family proteins in monocotyledonous plants (Otu1L) and mammals (Otub2), but lack the N-terminal UID domain known to interact with E2 enzymes. A ubiquitin-interacting motif (UIM) is found in Otu2 homologs. **b** In vitro deubiquitinase activity assay for the otubain domain of Otu2. The deubiquitinase activity against the tetra-ubiquitin substrates in Lys48-linked and Lys63-linked forms was examined by SDS-PAGE and immunoblot analysis using an anti-ubiquitin antibody. The in vitro deubiquitinase reaction was incubated for 30 min at 37 °C with 50 ng proteins. The C-terminal otubain domains of Otu2p, Otu2pC904S, and Otu2m proteins as well as the full-length Arabidopsis Otu1 (AtOtu1) proteins were produced in *E. coli* and purified by N-terminal 6x His-tag. The assay was repeated more than three times with technical duplicates.

*OTU2* encodes a divergent deubiquitinase (DUB) in the OTUB family^18^ (Fig. 3a and Supplementary Figs. 8, 9). To test whether *OTU2* encodes a catalytically active DUB, we performed in vitro DUB activity assays using the C-terminal OTU domain of Otu2. This domain (aa 731–1174) showed highly efficient cleavage of Lys48-linked tetra-ubiquitin into monomeric ubiquitin, comparable to Otu1 control proteins from *Arabidopsis* (AtOtu1), whereas the homologous domain of Otu2m (aa 731–1179) showed inefficient catalysis (Fig. 3b). Lys63-linked tetra-ubiquitin chains were not efficiently cleaved by AtOtu1 proteins, as previously reported^19^, nor by Otu2m proteins. In contrast, Otu2p proteins showed near-complete cleavage of Lys63-linked tetra-ubiquitin into monomers. Collectively, Otu2p and Otu2m were differentiated by both substrate preference and catalytic efficiency, and both deviated from the typical Lys48-preference of OTUB family proteins.

To determine whether DUB activity is required for the protective function of Otu2p, a non-catalytic form of the full-length Otu2p was generated carrying a Cys904Ser mutation that abolished the cleavage of Lys48- and Lys63-linked polyubiquitin chains (Fig. 3b). The expression of *cOTU2p*^*C904S*^ in *minus* cells failed to increase biparental cpDNA inheritance (Supplementary Fig. 10), suggesting that DUB activity is critical for the protective function of Otu2p.

This finding led to a search for ubiquitin-dependent mechanisms that might potentially affect cpDNA degradation and are regulated by Otu2p. Given that organellar DNA maintenance depends on imported proteins encoded by the nuclear genome, we hypothesized that Otu2p might regulate the trafficking of chloroplast-localized proteins. Two E3-ubiquitin-ligase pathways are known to target chloroplastic proteins for proteasome-mediated degradation in land plants: the first entails the participation of SP1 (*SUPPRESSOR OF PP1 LOCUS 1*), localized to the chloroplast envelope, which regulates chloroplast preprotein import by ubiquitylating translocons of the outer envelope membranes (TOCs)^20^; the second entails the participation of cytosolic CHIP (*CARBOXY TERMINUS OF HSC70-INTERACTING PROTEIN*), which targets preproteins directed to the chloroplast^21^.

We first asked whether the CHIP pathway affects cpDNA inheritance. We characterized two independent insertional mutant strains (*chip-1* and *chip-2*) that harbor deletions in the *C. reinhardtii* homolog of *CHIP* (Supplementary Fig. 11a, b). The *chip* mutants underwent normal gametogenesis and carried out normal cpDNA inheritance: 75–83% of the homozygous and heterozygous *chip* zygotes selectively degraded cpDNA, comparable to wild-type zygotes (Supplementary Fig. 11c), indicating that the CHIP pathway is not required for selective cpDNA degradation.

**Fig. 4:**
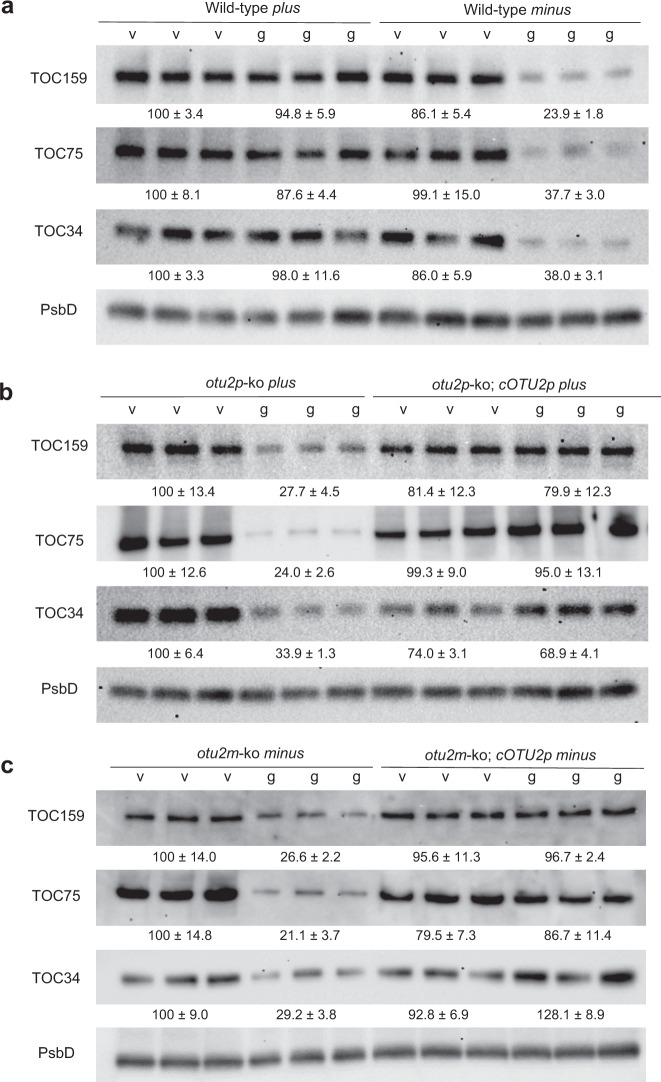
*OTU2p* prevents the reduction of TOC proteins during gametogenesis. The TOC159, TOC75, and TOC34 proteins were quantified by western blot using a protein extract from vegetative cells (v) and gametes (g) with PsbD as a gel-loading reference. Biological triplicates were loaded together. The average % TOC contents of triplicate samples relative to the left-most sample are shown below the blots (mean ± s.d.) **a** wildtype; **b** *otu2p*-ko; **c** *otu2m*-ko.

We next explored the SP1 pathway. Since an *SP1* mutant is not available in *C. reinhardtii*, we elected to examine the quantity of SP1-targeted proteins as a readout of SP1 activity. In *Arabidopsis*, SP1 targets the three primary components of TOC complexes: TOC34, TOC75, and TOC159^20^. Strikingly, levels of all three proteins were reduced by >60% in *minus* cells compared to vegetative cells during gametogenesis, whereas *plus* cells showed no significant reduction in TOC levels in gametes (Fig. 4a). This result suggests differential SP1 activities in *plus* vs. *minus* gametes.

To test whether the divergence between *OTU2p* and *OTU2m* is behind the mating type-specific difference in the TOC contents, *OTU2* knock-out strains were examined for their TOC quantity changes during gametogenesis*. otu2p*-ko gametes reduced TOC quantities down to 24–34% of vegetative levels (Fig. 4b), comparable to wild-type *minus* and *otu2m*-ko gametes (Fig. 4c). Furthermore, *cOTU2p* expression in either *otu2p*-ko or *otu2m*-ko gametes prevented the TOC level decrease during gametogenesis (Fig. 4b, c), indicating that *plus*-specific *OTU2p* drives differential TOC quantity between *plus* and *minus* gametes, presumably by interfering with SP1 and/or other ubiquitin-dependent mechanisms that regulate TOC levels.

### Otu2p interferes with proteasome-mediated protein degradation

**Fig. 5:**
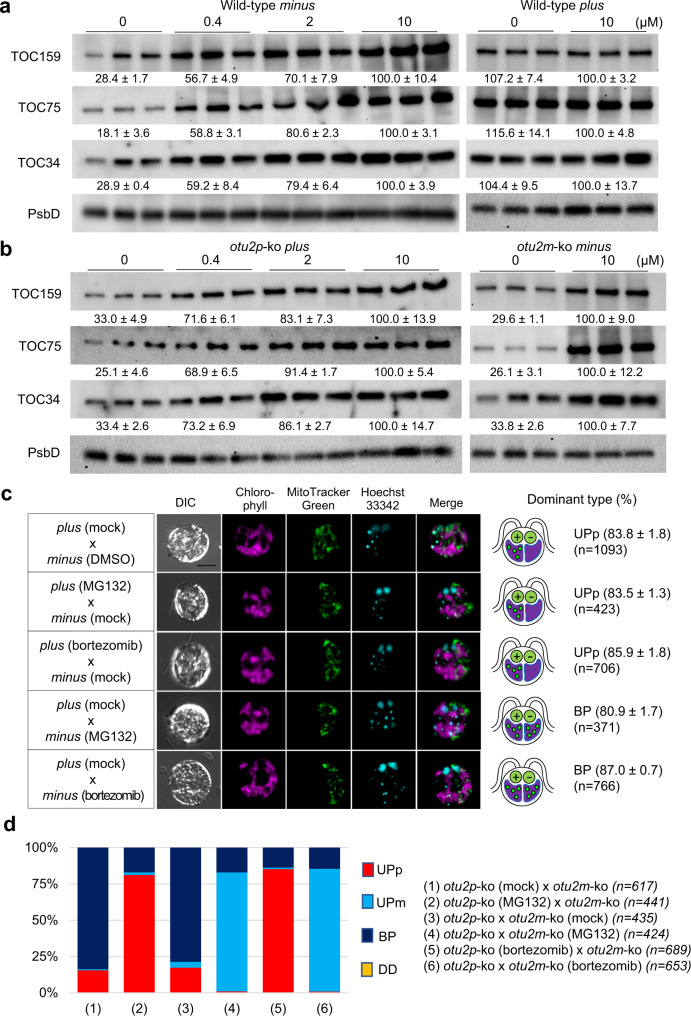
Unequal chloroplast import machinery between two chloroplasts drives selective cpDNA degradation in zygotes. **a**, **b** Proteasome-inhibitor treatment (MG132, 10 μM, 4 h) reversed TOC degradation in wild-type *minus* (**a**) and *otu2p*-ko (**b**) gametes. Gametes were incubated with MG132 at the indicated concentrations for 4 h and washed three times before mating. The TOC159, TOC75, and TOC34 proteins were quantified relative to the PsbD by western blot analysis. Values below the blot show the average % TOC contents relative to the samples treated with 10 μM MG132 (mean ± s.d.) Biological triplicates were loaded together. **c** Proteasome-inhibitor (MG132 or bortezomib) treatment protected *minus* cpDNA from degradation in wild-type zygotes. Differential interference contrast and overlaid fluorescent images of Hoechst 33342 (cyan)-stained zygotes with chlorophyll autofluorescence (magenta) were taken at 90 min after mating. *Minus* gametes were stained with MitoTracker Green to distinguish *plus*- from *minus*-derived chloroplasts. The percentage of the dominant cpDNA degradation pattern for each mating combination was calculated from three biological replicates (mean ± s.d.) Bar = 5 μm. “*n*” indicates the total number of zygotes examined. **d** Proteasome-inhibitor treatment established selective cpDNA degradation in *otu2*-ko zygotes. The average percentages of uniparental for *plus* (UPp), uniparental for *minus* (UPm), biparental (BP), and double degradation without cpDNA staining (DD) were calculated from three biological replicates. “*n*” indicates the total number of zygotes examined. Stacked bar graphs show four cpDNA degradation patterns, whose detailed values are provided in Supplementary Table 3.

Given that Otu2p is involved in regulating TOC profiles, we investigated its mechanism of action. First, we asked if proteasome-mediated degradation drives the decrease in TOC levels in gametes. Wild-type *minus* gametes treated with a proteasome-specific inhibitor, MG132, showed a full reversal of TOC content back to the levels of *plus* gametes in a dose-dependent manner (Fig. 5a). A full recovery of TOC content was also observed in the MG132-treated *otu2p*-ko gametes (Fig. 5b). In contrast, MG132 had little effect on TOC profiles in wild-type *plus* gametes (Fig. 5a), indicating that the presence of Otu2p effectively prevents the proteasome-mediated degradation of TOC proteins during gametogenesis and MG132 exposure is therefore redundant.

We next examined the effects of proteasome inhibition on cpDNA degradation in zygotes. When *minus* gametes were differentiated in the presence of MG132 and then mated, cpDNA degradation was spared. In contrast, the same pre-treatment in *plus* gametes had no effect on selective cpDNA degradation (Fig. 5c). We repeated the experiment with a second proteasome inhibitor, bortezomib, and confirmed the protective effect of proteasome inhibition on cpDNA (Fig. 5c). To evaluate the effect of proteasome inhibition independent of mating-type background, we examined otu2pm-ko zygotes where Otu2p is absent and all cpDNA is therefore unprotected. Treating *otu2p*-ko or *otu2m*-ko gametes with MG132 or bortezomib restored selective cpDNA degradation in >80% of zygotes, where the cpDNA of inhibitor-treated *plus* and *minus otu2*-ko gametes was protected while that of the untreated gametes was selectively degraded (Fig. 5d and Supplementary Fig. 12). These results demonstrate that the *plus*-specific Otu2p interferes with proteasome-mediated protein degradation in gametes, driving selective cpDNA degradation in chloroplasts with reduced TOC contents in the zygote.

## Discussion

Uniparental cpDNA inheritance, first reported in *C. reinhardtii* more than 60 years ago^10^, involves rapid elimination of cpDNA from *minus* gametes in young zygotes^11^. Genetically, the *MT*+ mating-type is dominant and drives preferential transmission of its cpDNA^9^. Similarly, in the smut fungus *Ustilago maydis*, the dominant *a2* mating-type drives selective transmission of its mitochondria^22^. In both cases, lack of the dominant mating-type leads to biparental inheritance (Fig. 2c; refs. ^15,22^), suggesting that the uniparental organelle inheritance evolved via selfish mechanisms that favor one’s organelles over the other.

In *U. maydis*, the *a2*-resident *LGA2* gene, encoding a putative E3-ubiquitin ligase localized to the mitochondrial surface, is required for *a2* dominance^22^. Ectopic expression of *LGA2* converts large mitochondria into smaller organelles, a phenomenon called mitochondrial fragmentation^23^, typically caused by reduced mitochondrial fusion. However, it remains unknown how Lga2 contributes to the discriminatory transmission of the *a2* mitochondria.

Our work identifies the Otu2p deubiquitinase as the *MT*+ -encoded protector of cpDNA, and shows that Otu2p interferes with ubiquitin-dependent mechanisms affecting the chloroplast envelope proteome. Since Otu2p lacks a transit sequence that would direct it into the chloroplast interior, other proteins that are cytoplasmic or accessible from the cytoplasm like the TOC proteins may also be protected by Otu2p activity. Further studies will be needed to identify the full pathway; here, TOC retention/degradation serves as a proxy for the operation of that pathway. Below we consider how translocon modification might participate in uniparental inheritance.

TOC content decreased in *minus* gametes but not in *plus* gametes expressing Otu2p. In land plants, various TOC complexes consist of multiple isoforms of TOC159 and TOC34 GTPases as preprotein receptors, exhibiting selectivity for their client proteins^24^, and numerous mechanisms are known to regulate the proteasome-mediated turnover of different TOC complexes to control chloroplast biogenesis, differentiation, and photosynthesis in response to stress and developmental signaling^20,25,26^. In *C. reinhardtii*, two TOC159 isoforms and a single TOC34 isoform are expressed in gametes. Possibly the TOC turnover mechanism induced in gametes may target specific isoforms of TOC complexes, and its interference by Otu2p may influence preprotein selectivity between *plus* and *minus* chloroplasts in the zygote, in which case differential TOC content might determine which cpDNA should be degraded in zygotes.

Young zygotes rapidly upregulate genes encoding organelle-targeted proteins, including DNA replication enzymes and single-strand DNA-binding proteins such as RecA^27^ (Supplementary Table 4), predicting major restructuring of organellar genomes at this juncture. Extensive nucleotide incorporation into cpDNA was also observed in young zygotes without increasing cpDNA copy number^28^, interpreted as repair synthesis to replace aberrant bases accumulated during gametogenesis under nitrogen limitation^29,30^. This repair likely involves recombination between redundant cpDNA copies, producing transient double-strand DNA breaks, which must be guarded against error-prone non-homologous interactions that may lead to DNA deletion^31,32^. Therefore, a failure or interference in safeguarding the recombinational repair process may lead to a catastrophic loss of cpDNA, in which case Otu2p, serving to retain intact TOCs and perhaps other outer envelope proteins, would be adaptive if it led to promoting error-free repair in zygotes and thus increasing cpDNA integrity.

Given the observed and predicted changes in the chloroplast proteome caused by Otu2p, the transmission of *plus* cpDNA may be fully committed at the gametic stage. On the other hand, the extent of *minus* cpDNA degradation does not seem to be determined at the gametic stage. It has been reported that mutation-inducing ultraviolet irradiation or 5-fluorodeoxyuridine treatment of *plus* gametes increased biparental cpDNA transmission and arrested the degradation of *minus* cpDNA^33–36^. Similarly, the *mat3* mutant, with a reduced number of cpDNA copies due to its small cell size, produced mostly biparental progeny in crosses of *mat3 plus* × wild-type *minus* but uniparental *plus* progeny in crosses of wild-type *plus* × *mat3 minus*^37^. These reports collectively suggest that *minus* cpDNA degradation is not predetermined and may be withheld when *plus* cpDNA copies and/or integrity are compromised and functional cpDNA levels drop below the threshold, a mechanism we can term the cpDNA checkpoint. The checkpoint assures that zygotic execution of cpDNA degradation is cognizant of and tempered by the cpDNA input from the *plus* chloroplast.

Our results add further evidence to support the existence of such a checkpoint. If gametic cpDNA were predetermined for degradation and Otu2p was required to block this degradation, then otu2p-ko zygotes, where both *plus* and *minus* cpDNAs are unprotected, would be predicted to show nonselective cpDNA degradation and high lethality. However, both *plus* and *minus* cpDNA were retained in >80% of the otu2p-ko zygotes (Fig. 2 and Supplementary Table 3).

We should note that the BP pattern of cpDNA degradation in otu2p-ko zygotes could be interpreted to indicate that Otu2p is directly required for cpDNA degradation; for instance, the *plus* gamete-specific nuclease (MDN) has been characterized as a candidate enzyme for zygotic cpDNA degradation^38^, and Otu2p might protect MDN in *plus* gametes. Under such a scenario, we would expect to see reduced cpDNA degradation in zygotes lacking Otu2p. However, 10.2–13.5% of the otu2p-ko zygotes still showed selective cpDNA degradation, comparable to 12.2% of the wild-type *plus* × *cOTU2p minus* zygotes (Fig. 2 and Supplementary Table 3), indicating no notable difference in cpDNA degradation capacity between the zygotes with and without Otu2p. Thus, Otu2p is apparently not required for cpDNA degradation.

**Fig. 6:**
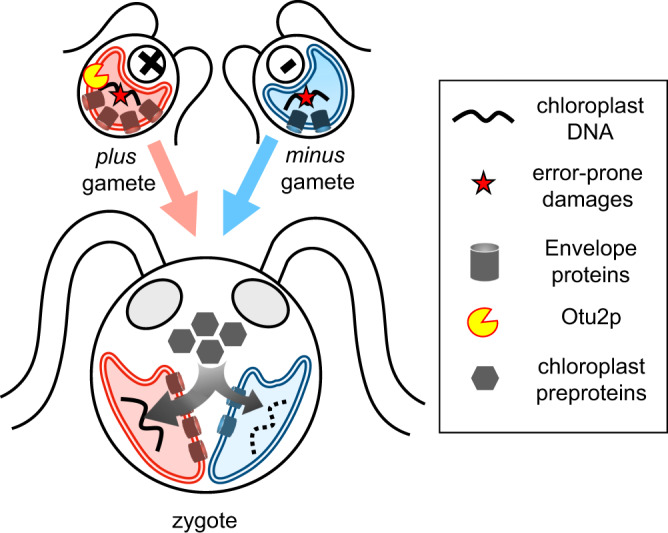
A model of the *OTU2p*-dependent mechanism for driving selective cpDNA degradation in zygotes. The model postulates gametes accumulating error-prone damages in cpDNA under nitrogen starvation. Mating-type-linked deubiquitinase *OTU2p* (yellow shape) prevents proteasome-mediated degradation of envelope proteins (barrel shape) including TOC translocase in *plus* gametes but not in *minus* gametes lacking Otu2p. This creates a visible disparity between *plus* and *minus* chloroplasts (double-lined cup shape), which leads to a biased flow of repair components including zygote-specific chloroplast-targeted preproteins (hexagons) between two chloroplasts combined in a zygote, triggering rapid cpDNA degradation (dotted wiggled line) in *minus* chloroplast delayed for importing repair safeguards. Therefore, the evolution of *OTU2p* would be adaptive for improving the quality of *plus* cpDNA and consequently for driving robust uniparental cpDNA inheritance.

Figure 6 shows a working model of the *MT*+-driven selective degradation of cpDNA. The first stage is gametogenesis, triggered by environmental stressors, during which the cells prepare for the repair of mutagenic modifications on organellar DNAs caused by stressors. Upon the fusion of *plus* and *minus* gametes distinguished by their chloroplast importing capacity, the *plus* chloroplast secures enough repair proteins and nucleotides for high-fidelity repair, while the *minus* chloroplast, lacking the necessary supply of DNA repair components, is vulnerable to complete degradation. When the *plus* and *minus* chloroplasts are instead equally endowed, as exemplified by otu2pm-ko zygotes, or when *plus* cpDNA is compromised, the competitive advantage of *plus* chloroplast would be erased, leading to a halt in *minus* cpDNA degradation. Indeed, without the wholesale degradation of *minus* cpDNA, cpDNA repair synthesis may be delayed due to an insufficient nucleotide pool. Once the two chloroplasts fuse, existing DNA damage would be recovered by genetic complementation and recombination-mediated repair between two cpDNA pools. Thereby, a critical evolutionary advantage is anticipated by a checkpoint coupled with “facultative” cpDNA degradation, serving as a rescue mechanism for irreversible accumulation of unfit mutations under strict uniparental transmission. Operation of this rescue mechanism predicts increased biparental organelle transmission in natural habitats exposed to mutagenic conditions^39^, which could rationalize the retention of organellar fusion during sexual mating^40,41^.

The mating-type linkage of *OTU2* in *C. reinhardtii* raises a question: Is this linkage associated with its protector role? *OTU2* homologs are found in the mating-type loci of other unicellular relatives of *C. reinhardtii* in the Metaclade-C of the core-Reinhardtina lineage (e.g., *C. schloesseri* and *Edaphochlamys debaryana*; Supplementary Table 5)^42^. By contrast, *OTU2* resides outside the mating-type loci in multicellular relatives in the TGV clade of the lineage, such as *Gonium pectorale* and *Volvox carteri*^43,44^, even though their mating-type loci are in many respects homologous to those of *C. reinhardtii*^45,46^. *OTU2* genes are also found in unicellular members of a related lineage, the Sphaeropleales (Supplementary Fig. 8), whose mating-type loci have not been identified.

The dominant *MID* gene involved in mating-type determination is found in all of the sequenced mating-type loci of the core-Reinhardtina^45,46^, but genes involved in many features of unicellular and multicellular sexual life cycles do not reside in the mating-type loci and are regulated in *trans*; hence the movement of *OTU2* out of the mating-type loci in the multicellular genera may or may not be relevant to its function. This shift does, however, disallow the occurrence of the sequence divergence observed between Otu2p and Otu2m in *C. reinhardtii*, a divergence presumably enabled by the suppression of recombination in the *C. reinhardtii* mating-type locus region^9^.

The role of *OTU2* in the sexual cycle of multicellular relatives of *C. reinhardtii*, if any, has not been determined. In the multicellular TGV clade, uniparental cpDNA inheritance has been reported in oogamous *V. carteri* and in *Gonium* species producing equal-sized gametes^47–49^. Interestingly, cpDNA degradation has been observed in *V. carteri* sperm before and during mating^50^. It is possible that differential expression of *OTU2* without sex-specific sequence divergence may differentiate gametic chloroplasts according to their mating type. Alternatively, the Otu2-dependent mechanisms may have been supplanted by other modes of uniparental inheritance in the multicellular lineages. Experiments that evaluate the effects of *OTU2* knockouts in these lineages are clearly warranted.

In animals, mitochondrial genomes show high mutation rates, in part due to strict uniparental transmission, rendering recombinational repair more difficult. This condition demands genome quality-control mechanisms selecting the best-fit mitochondria in the female germline^51^. A recent study of fly oogenesis reported that developing eggs downregulate the mitochondrial fusion protein Mitofusin via proteasome-mediated degradation, leading to multiple small mitochondria^52^. This reduces genomic redundancy per mitochondria, which allows selection against faulty genome copies. Given the apparent similarity of Lga2 action in *U. maydis* for promoting mitochondrial fragmentation^23^, the Lga2-dependent uniparental mitochondrial inheritance in *U. maydis* may have evolved by coopting mitochondrial genome quality-control mechanisms.

Our work describes a ubiquitin-dependent mechanism to distinguish two chloroplasts in a cell. Given that TOC regulation serves as a critical mechanism to control chloroplast functions, Otu2p’s interference with the TOC turnover resembles the Lga2-dependent mitochondrial inheritance mechanism in tweaking the existing organelle quality-control mechanisms dependent on the ubiquitin-proteasome system. Hence our results propose a common theme for the evolution of uniparental organelle inheritance in two simple eukaryotes from disparate lineages.

## Methods

### Strains and culture conditions

Wild-type (WT) *plus* (CC-125), WT *minus* (CC-621), nicotinamide auxotroph *nic7 plus* (CC-85), erythromycin-resistant *plus* (eryR, CC-333), *nic7 minus* eryR (CC-2663), cell wall-less *cw15 plus* (CC-400), and CLiP mutants, *chip-1 and chip-2 minus* (LMJ.RY0402.080594 and LMJ.RY402.209732^53^) were obtained from the Chlamydomonas Resource Center (https://www.chlamycollection.org/). The *cOTU2p*- or *cOTU2m*-expressing *minus* strains, and *otu2p*-ko and *otu2m*-ko strains, which were generated in this study, are available from the Chlamydomonas Resource Center with the following accession numbers: CC-5776 *cOTU2p minus*; CC-5778 *cOTU2m minus*; CC-5779 *otu2p*-ko *plus*; CC-5780 *otu2m*-ko *minus*.

The *cOTU2p* or *cOTU2m* transgenes were introduced into the *otu2p*-ko or *otu2m*-ko strain by sexual crosses. WT *plus* eryR (CC-333), *nic7 minus* eryR (CC-2663), *nic7 minus* specR (spectinomycin-resistant, CJU10^54^), and WT *plus* specR (CJU10-J1, sexual progeny of CJU10) were used for genetic analysis of cpDNA inheritance. A paromomycin-resistant *plus* eryR strain (T-par#5-40) was used for large-scale screening of transformants generated by glass bead-based transformation of the *aph*VIII plasmid with CC-333 as a host strain. Cells were maintained under medium light (50 μmol photons m^−2^ s^−1^) at 23 °C on Tris-acetate phosphate (TAP) medium containing 1.5% Bacto agar. Nitrogen-free TAP (N-free TAP) medium was prepared by omitting nitrogen. The following antibiotics (concentrations) in the TAP medium were used for screening of the colonies: spectinomycin/erythromycin (100 µg mL^−1^), hygromycin (25 µg mL^−1^), and paromomycin (10 µg mL^−1^). The proteasome inhibitors MG132 (1748/5, Tocris Bioscience) and bortezomib (S1013, Sigma) were dissolved in dimethyl sulfoxide and used at a concentration of ≤10 µg/mL.

### Molecular analysis of *OTU2p* and *OTU2m*

To identify the BAC clones harboring the 165-kb region unique to the *MT*+ locus, the *C. reinhardtii* genome browser (available at https://phytozome.jgi.doe.gov/pz/portal.html) was scanned on the track of BLASTN-aligned BAC-end reads within chromosome 6:600,000–792,600. Three BAC clones (33d13, 18p21, and 33o16) were selected for the transformation of *minus* strains. A full-length OTU2p-A genomic clone was obtained from the StuI-AatII 5.9-kbp fragment of the 33d13 clone. The OTU2m genomic clone was isolated as an 8-kbp EcoRI fragment from the lambda phage clone BJ2, as reported by Ferris et al.^15^.

To define the OTU2p transcript structure, the 253-bp OTU2p cDNA fragment amplified by the F1 × R1p primer pair was used to screen plaque lifts of a cDNA expression library in Uni-ZAPXR (Stratagene, La Jolla, CA), prepared from 60-min zygotic poly(A) + RNA, in accordance with the procedures described by Ferris et al.^15^. The largest transcript consisted of 1709 bases, including 892 bases at the 3′-UTR (untranslated region). To identify the 5′-end of OTU2p, 5′-cDNA rapid amplification of cDNA ends was performed following the manufacturer’s protocol (Gibco BRL) using the reverse gene-specific primers R14 and R16, which revealed a 410 base-long 5′-UTR with two introns. The OTU2p transcript consisted of 4827 bases, as confirmed by PCR-based cloning of four fragments combining more than one exon with the F13 × R9, F16 × R17, F7 × R1, and F2 × R12 primer pairs. The same exon-intron borders were confirmed for the OTU2m transcript. *OTU2p* and *OTU2m* expression levels stay relatively constant through life cycle, and show no significant difference between the two alleles, consistent with De Hoff et al.^16^. Sequence information of the OTU2p-A and OTU2m genes is available from the GenBank sequence database (OL770090 for OTU2p-A, OL770091 for OTU2m).

To construct a hemagglutinin-tagged OTU2 containing the coding segment of OTU2p (cOTU2p), the 1187-bp EcoRI-NotI fragment from EcoRI-tagged F16 × R5 PCR products, 2852-bp NotI-KpnI fragment from the OTU2p genomic clone, and 823-bp KpnI-EcoRI from F17 × EcoRI-tagged R12 PCR products were ligated into the EcoRI site of a pBS-based vector containing the hsp70Apro::rbsS2pro::[EcoRI]::3xHA epitope tag::rbcS2 terminator. To generate the Cys904Ser mutation, the 873-bp HindIII-EcoRI fragment of the OTU2p cDNA clone was subcloned and mutagenized with the C904S-F and -R primers. The mutagenized HindIII-EcoRI clone was ligated with a HindIII-EcoRI double-digested AR:cOTU2p-HA3× plasmid. All primer sequences used in this study are provided in Supplementary Table 6.

Real-time (RT)-PCR analysis was conducted to examine the expression levels of truncated OTU2p-B/C/D/F/G with allele-specific primer pairs, but their expression was undetectable. Available 454 transcript assembly and RNA-seq reads were examined for OTU2p transcripts, but none were the truncated forms, indicating that if the truncated OTU2p copies were expressed at all, it was below the detection limit.

### Generation of *otu2p*-ko and *otu2m*-ko strains

Purified recombinant Cas9 proteins (ToolGen, Inc.) were used to prepare the OTU2-targeting guide RNA-Cas9 protein complex. Two guide sequences targeting *OTU2* loci at *MT*+ and *MT−* were designed with the Cas-Designer tool (http://www.rgenome.net/cas-designer/) for knock-out experiments. All knock-out strains used in this study were constructed by targeting the 5′-CCCGAGGCGCATCAACTGGAATC-3′ sequence of exon 4 of *OTU2p* (1272–1294 bases) or *OTU2m* (978–1000 bases). Guide RNA was transcribed in vitro using the MEGAshortscript™ T7 Transcription Kit (Ambion) and purified by phenol:chloroform extraction and ethanol precipitation. Purified RNA was quantified by spectrometry.

To generate target-specific insertional knock-out mutants using the RNA-Cas9 protein complex, 5 × 10^5^ cells were transformed with the Cas9 protein (200 μg) in storage buffer (20 mM hydroxyethyl piperazine ethane sulfonic acid, pH 7.5, 150 mM KCl, 1 mM dithiothreitol [DTT], and 10% glycerol) mixed with in vitro transcribed sgRNA (140 μg), dissolved in nuclease-free water, and incubated for 10 min at room temperature. The cells were then transformed with the GeneArt^®^ Chlamydomonas Engineering Kit (Thermo Fisher Scientific), in accordance with the recommended protocol, and a Gene Pulser Xcell™ Electroporation System (Bio-Rad Laboratories). After transformation, the cells were incubated for 12 h and either harvested for genomic DNA (gDNA) extraction or immediately diluted, and ~2000 cells were plated on a TAP medium containing 25 μg mL^−1^ of hygromycin and 1.5% agar to obtain insertional knock-out transformants for further investigation. Five or ten colonies were picked and further analyzed.

To isolate gDNA, harvested cells were resuspended in microprep buffer containing 2.5× extraction buffer (0.35 M sorbitol, 0.1 M Tris/HCl, pH 7.5, and 5 mM ethylenediaminetetraacetic acid), 2.5× nuclei lysis buffer (0.2 M Tris/HCl pH 7.5, 0.05 M ethylenediaminetetraacetic acid, 2 M NaCl, and 2% (w/v) cetrimonium bromide), and 5× 5% *N*-lauroylsarcosine, and incubated for 2 h at 65 °C. The gDNA was extracted with chloroform:isoamyl alcohol (24:1) and precipitated with isopropanol. Southern blotting was performed as previously described^17^. To verify the targeted-insertion events, the Cas9-target regions were PCR-amplified with the ko-F1 × ko-R1p or ko-R1m primer pair. The PCR products were sequenced using the Sanger method. Verification of the knock-out procedure is summarized in Supplementary Fig. 6.

### Genetic analysis of cpDNA inheritance

BAC clone-based transformation typically yields <10% of transformants with a full-length gene, considering the lengths of the *OTU2* and *EZY2* genes (7.1 and 7.5 kb, respectively). To test the “protector” function of the BAC clones harboring the 165-kb *MT*+ region, at least 96 *minus* transformants were collected and examined for increased transmission of *minus* chloroplast markers (specR or eryR). Assuming a mating efficiency of 75%, about 100–200 zygotes were plated on the selection plate to kill unmated *plus* and *minus* parental cells. Following the zygote-maturation and germination protocol, surviving zygotes were considered as the progeny inheriting biparental or uniparental-*minus* cpDNA. The transgenic *minus* strains that showed increased zygote survival were reanalyzed by examining individual zygotes. Fifteen BAC-transformed strains in Supplementary Table 1 were selected for reanalysis. Between 43 and 123 individual zygotes were collected from the mating of these 15 eryR strains and a *plus* specR strain (CJU10-J1).

For each OTU2-engineered construct, at least 48 transgenic strains were collected using the *minus* specR strain, of which 15–16 strains were selected by detectable transgene expression at the transcript level for further analysis. Mating of the transgenic strains was done with a *plus* eryR strain (T-par#5-40), harboring a paromomycin-resistant nuclear marker (parR). The isolated zygotes were transferred to plates containing spectinomycin or erythromycin or both to quantify the uniparental or biparental progeny.

### Gametogenesis and mating

To induce gametogenesis, cells were scraped from a week-old plate and resuspended in N-free TAP to a final concentration of 5 × 10^7^ cells mL^−1^. After incubation for 3–5 h in the light, equal numbers of *plus* and *minus* gametes were mixed and allowed to mate. To quantify gametic activity, the mating efficiency was calculated at 1.5 h after mating based on the percentage of gametes engaged in the formation of quadriflagellate cells.

### Fluorescence microscopic analysis

To observe the degradation patterns of the chloroplast nucleoids, zygotes were stained with either 1:500 diluted SYBR green I (Invitrogen) or 100 μM Hoechst 33342 (Invitrogen) for 10 min and observed under GFP (bandpass 480/20) or Y5 (bandpass 620/60) filters. To distinguish *plus* and *minus*-derived chloroplasts, *minus* gametes were prestained with 100 μM MitoTracker Green FM (Invitrogen) for 30 min before mating. The cpDNA degradation pattern was quantified in at least 150 zygotes in biological triplicates.

### Quantitation of chloroplast genome copy number

To quantify chloroplast genome copies, total gDNA was extracted from cells under given conditions using the phenol/chloroform-based method described above. Quantitative-PCR analysis was performed using total gDNA and primers specific for two chloroplastic loci (*psbB* and *psbD*) and two nuclear loci (*RACK1 and cblp*). The cpDNA copy number is typically about 100, thus total gDNA preps were carefully diluted to ensure that the Cq (quant cycle) values were between 17 and 30. Copy numbers of *psbB* and *psbD* were calculated relative to *RACK1* or *cblp* as a reference single copy locus. Relative quantity estimates were comparable between the RACK1-based and cblp-based results; therefore, only the RACK1-based results are provided.

### Immunoblot analysis

To confirm transgene-derived Otu2 proteins, we performed immunoblots using antibodies against epitope HA (12CA5, Santa Cruz) but could not detect Otu2-specific signals. We also raised polyclonal antibodies against the bacteria-expressed C-terminal Otu2p protein and the N-terminal and C-terminal peptides (69-GLRSGSNTYDSRTAC and 1000-CPPRDDPAAYRGGQR) in rabbit (Genscript) but again failed to detect the Otu2 protein in immunoblots or in situ immunofluorescence hybridization.

To quantify translocons of the outer chloroplast membrane (TOC) contents, total protein extracts were prepared from 1 × 10^7^ cells, which were washed once, collected by centrifugation at 4000×*g* for 3 min, and frozen in liquid nitrogen. Subsequently, an equal volume of boiled 2× extraction buffer was added (100 mM Tris, pH 6.8, 200 mM DTT, 4% sodium dodecyl sulfate [SDS], and 20% glycerol) to each frozen cell pellet, and the mixture was immediately boiled at 95 °C for 10 min followed by centrifugation at the maximum speed (16,000 × *g*) for 10 min. Each lane was loaded with 3–5 μL of the protein extracts corresponding to 5 × 10^5^ cells. Proteins were separated by polyacrylamide gel electrophoresis (PAGE) with 4–20% SDS gels (Bio Basic) and transferred to polyvinylidene fluoride membranes (Immobilon-P, Millipore), which were blocked with 1% nonfat dry milk in TBST buffer (150 mM NaCl, 0.1% Tween-20 in 20 mM, Tris, pH 7.6) for 1 h, followed by incubation for 2 h at room temperature with the following antibodies: anti-TOC34 *Arabidopsis* (1:20,000, Agrisera AS07-238, detecting a major product at 48 kDa), anti-TOC75 pea (1:10,000, gift from Enrico Shleiff, detecting a major product at 75 kDa and nonspecific product at 35–48 kDa), or anti-TOC159 pea (1:10,000, gift from Bettina Bolter, detecting a major product just below 180 kDa and minor product at 75–100 kDa, the latter being a likely proteolytic fragment of the full-length TOC159 protein since the two products were consistently in the same ratio), as well as anti-PsbD pea (1:20,000, Agrisera AS06-146, detecting a single product at 25–35 kDa), as an internal control in TBST buffer containing 1% nonfat dry milk. After extensive washing with TBST, the blots were incubated with horseradish peroxidase (HRP)-conjugated goat anti-rabbit antibody (diluted 1:5000, Bio-Rad 5196-2504) in TBST containing 1% nonfat dry milk for 1 h, then extensively washed with TBST and treated with Luminata™ Western Chemiluminescent HRP Substrate (Millipore) as recommended by the supplier. Luminescence was imaged using the ChemiDoc™ MP Imaging System (Bio-Rad) with an exposure time that did not saturate any pixel of the collected 16-bit image. Quantification of the protein bands was performed using Image Lab software 6.1 (Bio-Rad). Quantities of each TOC protein were estimated by the total intensities of each TOC protein band relative to the intensity of the PsbD protein, a reference chloroplast protein. Images were obtained with the “optimal” setting directly from the 16-bit raw data. The full blots of Fig. 4a are included in Supplementary Fig.13, which confirm the specificity of the antibodies used in this study.

### In vitro DUB activity assay

To obtain His-tagged OTU proteins, the full-length coding region of AtOtu1 and coding sequences of the C-terminal catalytic domains of Otu2p (aa 731–1174) and Otu2m (aa 731–1179) were PCR-amplified from cDNAs prepared from total RNA extracts of *Arabidopsis* leaves or *C. reinhardtii* cells using Phusion High-Fidelity DNA Polymerase (New England Biolabs). The amplified fragments were cloned in-frame into the pET28a plasmid (EMD Millipore). Recombinant proteins were expressed in *E. coli* BL21 (DE3) cells (Novagen) and purified by affinity chromatography using Ni-NTA Agarose (Qiagen).

To characterize the DUB activity of Otu2 proteins, the in vitro DUB assay was performed using purified 6x His-AtOtu1, -Otu2p^[731-1174]^, -Otu2p^C904S[731-1174]^, or -Otu2m^[731-1179]^ proteins (50 ng estimated by Coomassie-blue staining of the purified proteins), as previously described^55^. In each set, 50 ng of purified proteins (estimated by Coomassie-blue staining) were mixed with 250 ng of tetra-ubiquitin substrate linked either by Lys48 or Lys63 (Boston Biochem) in 20 μL of reaction buffer (150 mM NaCl, 0.5 mM DTT, 20 mM Tris, pH 8.0) and incubated for 30 min at 37 °C. The reactions were stopped by heating after the addition of 2× SDS-PAGE sample buffer. The DUB activity against the tetra-ubiquitin substrates was examined by SDS-PAGE and immunoblot analysis using an anti-ubiquitin antibody (Santa Cruz Biotechnology).

### OTUB family phylogenetic analysis

To assess the diversification of OTU2 in the OTUB family DUBs, OTUB family members were collected by HMMsearch (v3.1b2) of the Peptidase_C65 domain profile (PF10275) against the selected genome-wide protein collections, mainly from Viridiplantae, with an *E*-value of E-05 as the per-domain inclusion threshold. The collected OTUB protein sequences were aligned together with selected OTUD sequences as outgroups using the MAFFT algorithm. The resulting aa alignment was manually inspected and trimmed to generate the final alignment of the OTUB catalytic domain (51 sequences with 277 informative sites; Supplementary Fig. 9b). Phylogenetic reconstruction was performed by the maximum-likelihood method using IQ-TREE. The LG + I + G4 model was chosen according to Bayesian Information Criteria. IQ-TREE yielded a strongly supported consensus unrooted tree with Ultrafast bootstrap test score with the “-bb 2000 -bi 500” option (Supplementary Fig. 8). An Ultrafast bootstrap score of 95% was considered highly significant, as recommended by the software authors.

### *OTU2* genetic linkage analysis among chlorophycean algae

To predict which genome scaffolds are linked to the mating-type loci, we first searched *MTD1* and *SAD1* that are known to reside in the mating-type loci in Volvocine algae including *Chlamydomonas reinhardtii* and *Volvox carteri*. The presence of *OTU2* with *MTD1* or *SAD1* homolog in the same scaffold suggests the genetic linkage of *OTU2* to the mating-type loci. Of seven genomes examined, no *OTU2* homolog was found in the *Chlamydomonas eustigma* genome. Except *Dunaliella salina*, we were able to determine the mating-type linkage of OTU2 (Supplementary Table 5).

### Reporting summary

Further information on research design is available in the Nature Research Reporting Summary linked to this article.

## Supplementary information

Supplementary Information

Reporting Summary

## Data Availability

All data generated or analyzed during this study are included in this published article and its supplementary information file. Source data are provided with this paper.

## Source data

Source Data
