# Supplementary material for: Sex-linked deubiquitinase establishes uniparental transmission of chloroplast DNA
Source: Nat Commun. 2022 Mar 3;13:1133. doi: 10.1038/s41467-022-28807-6 (PMC8894339; doi:10.1038/s41467-022-28807-6)
Supplement: Supplementary file 1 — Supplementary Information [file 41467_2022_28807_MOESM1_ESM.pdf]

Supplementary Materials for

Sex-linked deubiquitinase establishes uniparental transmission of chloroplast DNA

Sunjoo Joo[1], Thamali Kariyawasam[1], Minjae Kim[2], EonSeon Jin[2], Ursula Goodenough[3], and Jae-Hyeok Lee[1*].

Correspondence to: jae-hyeok.lee@botany.ubc.ca

This PDF file includes:

Supplementary Figs. 1 to 13
Supplementary Tables 1 to 6
Supplementary References 1 to 9

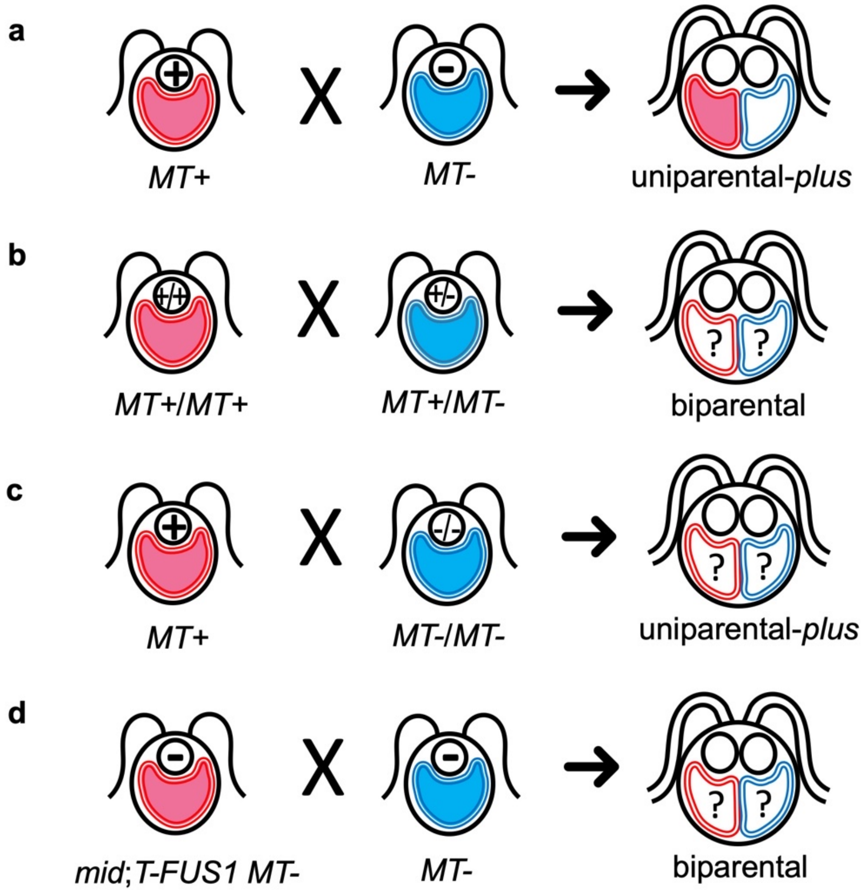

Supplementary Figure 1. The dominant control of *MT*+ locus for the uniparental inheritance of cpDNA in *C. reinhardtii*. The following genetic experiments postulate two functions genetically linked to *MT*+ for the uniparental inheritance of cpDNA: a "*protector*" function that protects cpDNA from degradation in zygotes and a "*destroyer*" function (elusive) that controls the onset of cpDNA degradation in early zygotes. (**a**) WT mating between *plus* and *minus* gametes produces uniparental progeny predominantly inheriting *plus*-derived cpDNA. *Minus*-derived cpDNA is completely degraded in zygotes (indicated by empty chloroplasts) *(1)*. (**b**) Mating between *MT*+/*MT*+ and *MT*+/*MT*- diploid gametes produces biparental progeny receiving a mixture of both cpDNAs *(2)*, suggesting that the protection function is linked to *MT*+. However, whether the *minus* cpDNA from *MT*+/*MT*- gametes escapes the zygotic degradation remains unknown (indicated by question marks in the zygotic chloroplasts). (**c**) Mating between haploid *plus* (*MT*+) and diploid *minus* homozygous at mating-type loci (*MT*-/*MT*-) produces progeny predominantly receiving *plus*-contributed cpDNA, suggesting that an imbalance in the amount cpDNA cannot override the *MT*+-dependent "protector" function *(2)*. (**d**) Mating between WT *minus* gametes and *mid*-null *MT*- mutants (thereby behaving as *plus)* harboring *T-FUS1* (allowing the fusion with WT *minus* gametes) produces biparental progeny *(3)*. This phenotype has been explained by the *MT*+-encoded "*destroyer*" function that is absent in *MT*-.

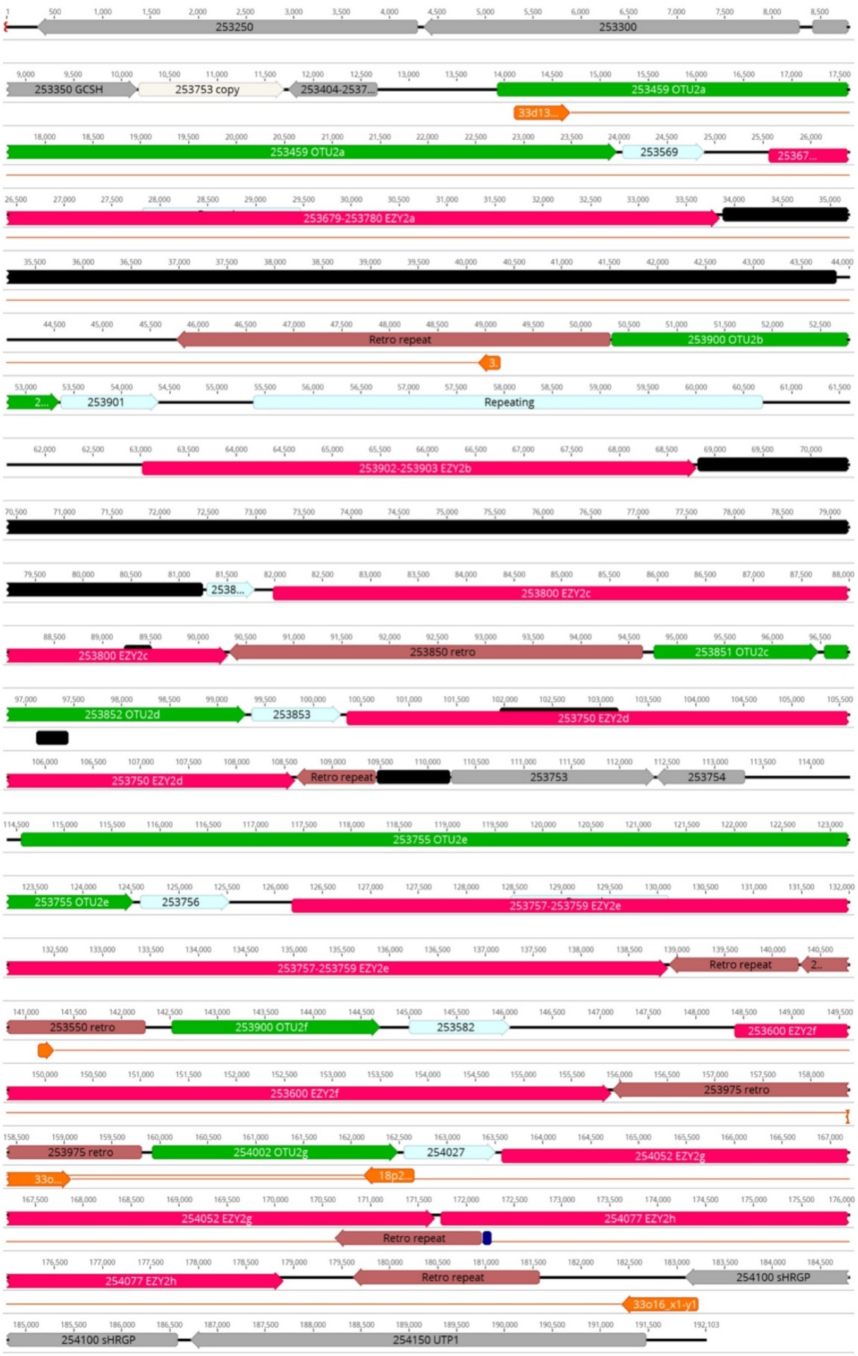

Supplementary Figure 2. The gene content of the *OTU2p*/*EZY2* cluster in the R-domain of *MT*+. The *MT*+ R-domain corresponds to 600–792.5 kb in chromosome 6 of v.236 genome at Phytozome (https://phytozome-next.jgi.doe.gov). At least seven copies of *OTU2p*/*EZY2*-containing 16-kb fragments were found, as reported earlier *(3)*. Orange bars indicate the predicted locations of the three BAC clones used in this study. Gene annotations: green, *OTU2p*-A-F copies; magenta, *EZY2* copies; black, sequencing gaps; light blue, the repeating elements located between *OTU2p* and *EZY2* copies; brown, retrotransposon-like repeating elements; gray, genes outside the *OTU2p*/*EZY2* repeats.

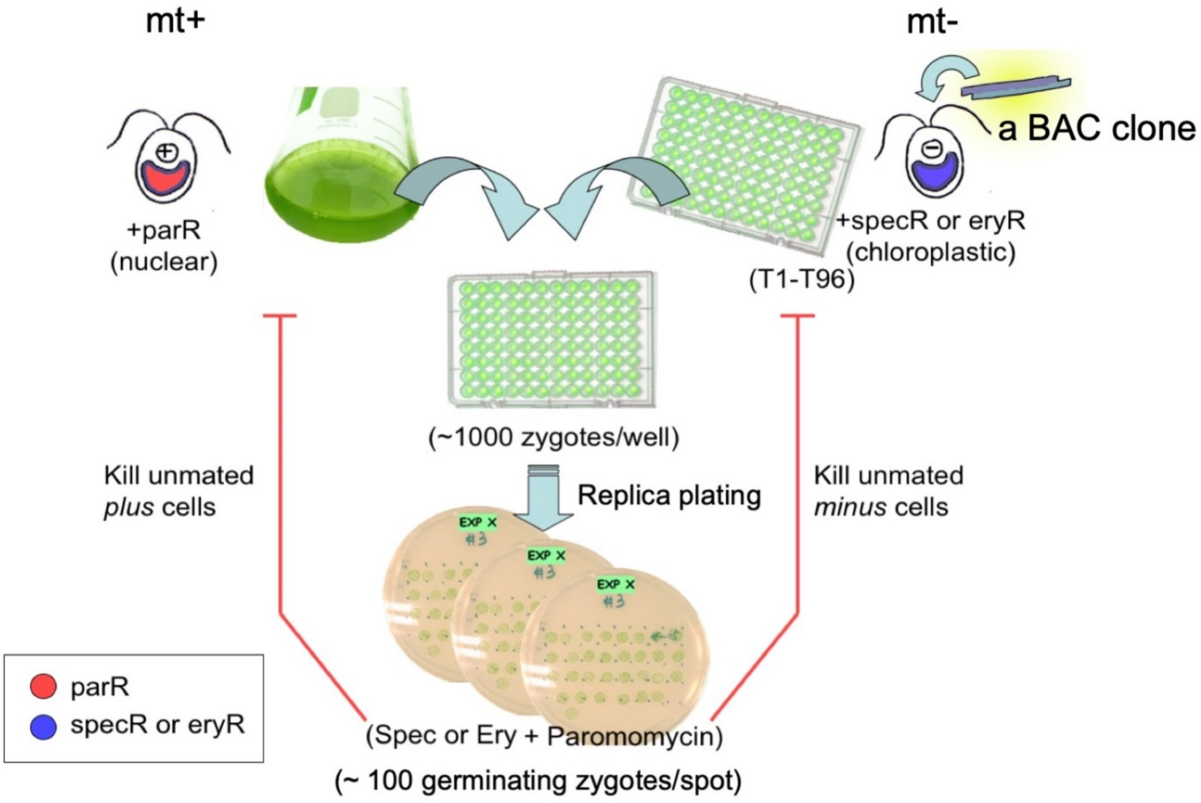

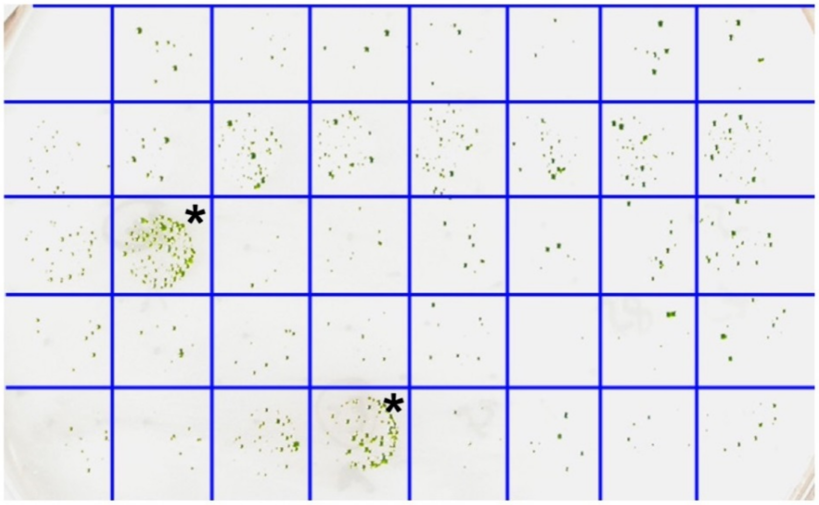

Supplementary Figure 3. An experimental scheme for testing the "*protector*" function. Experimental details are found in Methods. Below the diagram is an example of the screening result, where two of 40 spots show an increase in the number of surviving zygotes (asterisks).

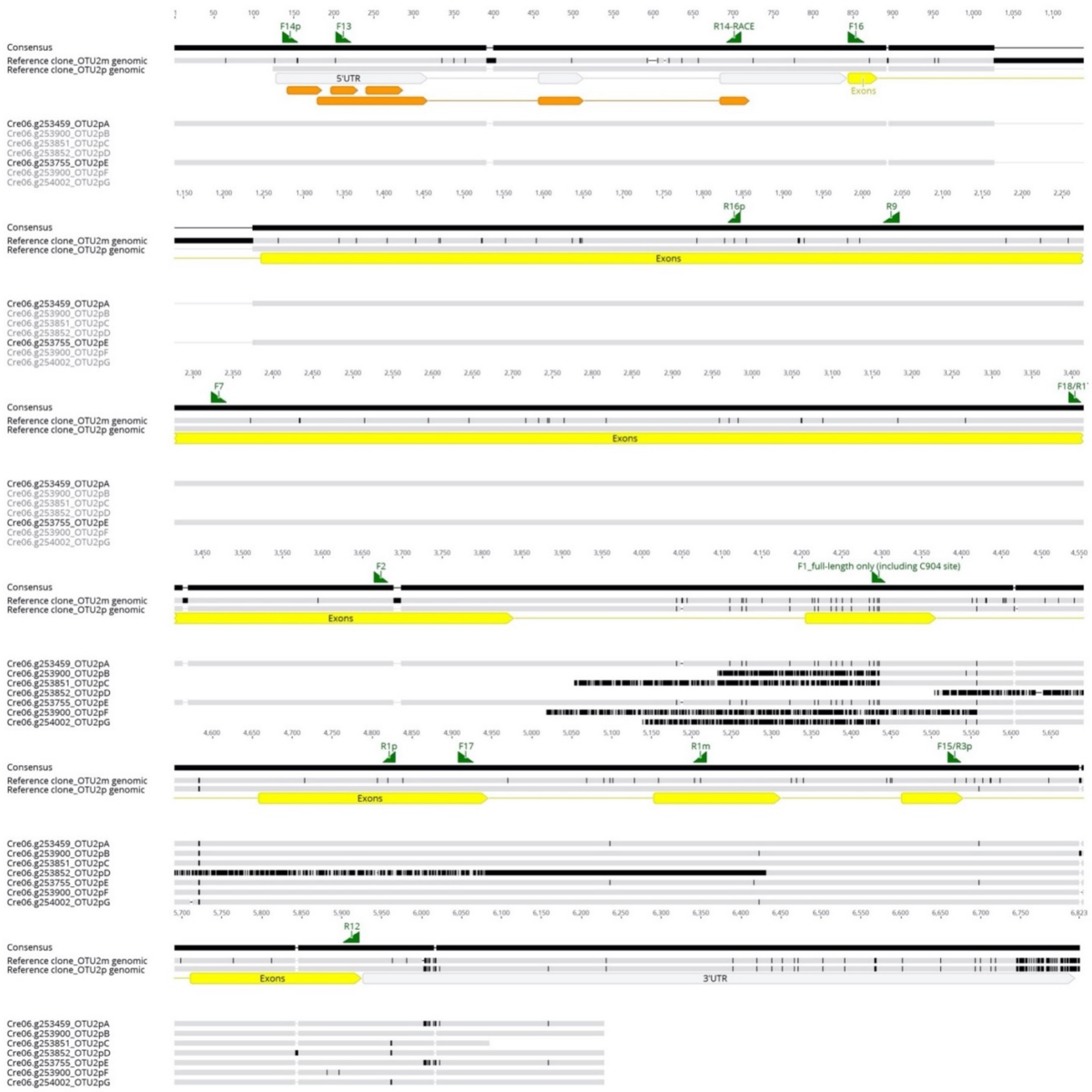

Supplementary Figure 4. *OTU2* gene structure and polymorphisms among the *OTU2* alleles. Reference *OTU2p* and *OTU2m* sequences were obtained from the BAC clone 33d13 and the lambda phage clone BJ2 *(3)*, respectively. *OTU2p* and *OTU2m* exon-intron structures were confirmed by cDNA cloning. Nine exons were found with two introns in the 5'-UTR. The *OTU2p* 5'-UTR also contained four open reading frames (orange) that terminate before the ATG start codon of the *OTU2* main open reading frame (yellow). *OTU2p*-A/E copies contain a full-length gene structure, whereas *OTU2p*-B/C/D/F and -G copies are truncated by transposon-like repetitive sequences (indicated by dense black vertical lines between 3900 and 5300 bp), including only the upstream C-terminal segment. None of the truncated *OTU2p* copies include the Cys904 codon, essential for otubain catalysis. Sequences are shaded in gray, where black vertical lines indicate differences among *OTU2* copies. Primers used in this study are indicated by green wedges.

a

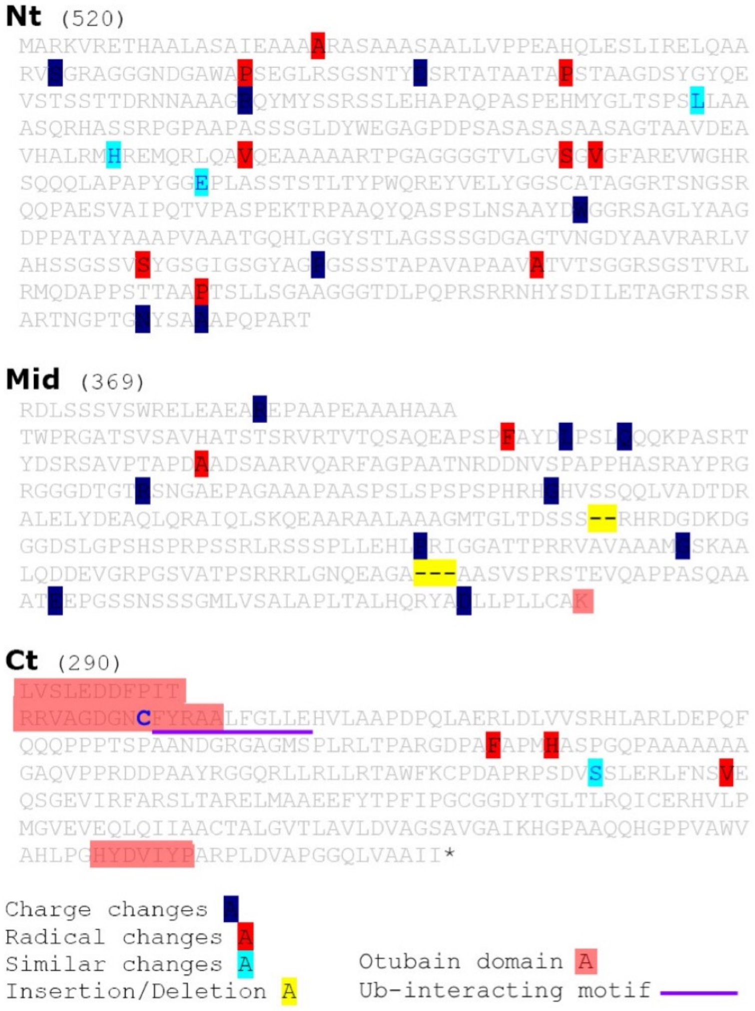

b

Changes	Charged	Radical	Similar
Nt	1.3%	1.7%	0.6%
Mid	**2.4%**	**1.9%**	0.0%
Ct	0.0%	1.0%	0.3%

Supplementary Figure 5. Protein sequence comparison between Otu2p and Otu2m. (**a**) A total of 39 changes were found in 1137 aa. Highlighted sequences indicate aa changes between Otu2p and Otu2m in charged, radical, similar, and insertion/deletion categories by navy, red, light blue, and yellow colors, respectively. Cys904, a critical cysteine for DUB activity, is marked in bold blue in the conserved OTU domain located at the beginning and end of Ct (light red shades). In total, 19, 16, and 4 aa positions differed between Otu2p and Otu2m in the N-terminal, middle, and C-terminal segments, respectively. (**b**) Summary of aa substitution rate per aa category in the three segments.

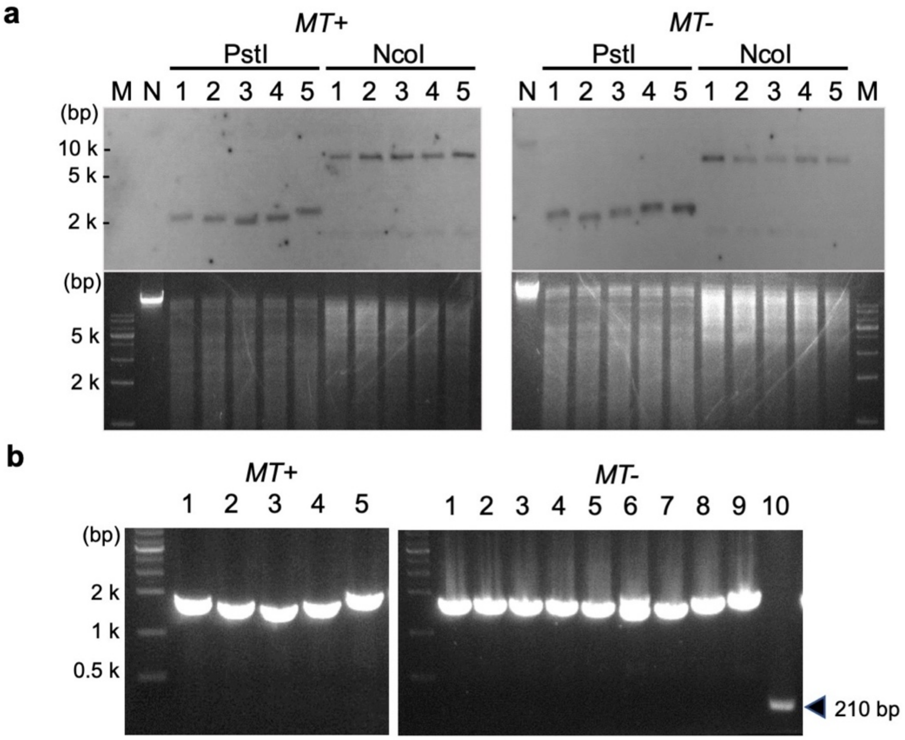

c

Cross	Mating efficiency at 1.5 h	UP%
WT *plus* x WT *minus*	82.6%	70%
otu2p-ki_2 x WT *minus*	73.5%	28%
otu2p-ki_3 x WT *minus*	76.6%	22%
otu2p-ki_4 x WT *minus*	75.47%	42%
otu2p-ki_5 x WT *minus*	74.6%	40%
WT *plus* x *otu2m*-ki_1	77.5%	68%
WT *plus* x *otu2m*-ki_2	75.47%	75%
WT *plus* x *otu2m*-ki_3	74.6%	78%
WT *plus* x *otu2m*-ki_4	73.26%	61%
WT *plus* x *otu2m*-ki_5	81.3%	71%

Supplementary Figure 6. CRISPR-Cas9-mediated generation of targeted knock-out mutants of *OTU2p* and *OTU2m*. (**a**) Southern blotting confirmed the single-locus insertion of *aphVII*, conferring hygromycin resistance. Wild-type gDNA did not hybridize with the aphVII probe. (**b**) gDNA amplification by PCR using the primer pair OTU2-ko-F1 and OTU2-ko-R1p/OTU2-ko-R1m (Supplementary Table 6*),* and sequencing confirmed insertion of a single copy of *aphVII* (1361 bp) at the 1-CCC/GAG-6 base in the targeted sequence in *otu2p* knock-out mutants #2–5 and all five *otu2m* knock-out mutants. Uninterrupted *OTU2p* or *OTU2m* copies should produce 210-bp products (#10 in *MT*- samples). The lack of 210-bp products indicates the absence of a full-length *OTU2* allele. *OTU2p* knock-out mutant #1 has an insertion at the last site of the targeted sequence (~ATC-23/GCT). The different band sizes are due to 5–101-bp deletions at the insertion sites. (**c**) The mating activities of *otu2p*-ko #2-#5 and *otu2m*-ko #1-#5 and selective cpDNA degradation in zygotes following mating with WT gametes were examined. All *otu2p*-ko mutants showed a significant reduction in the UP rate at 1.5 h after mating. For each sample, a total of 100 zygotes were examined.

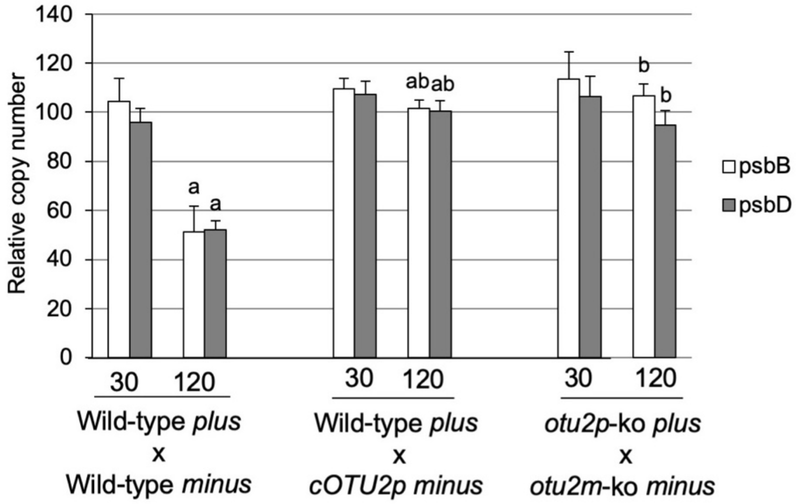

		Wild-type 120 min	cOTU2p 120 min	otu2-ko 120 min
(a)	30 min zygotes	0.0005	0.0118	0.4338
(b)	wild-type 120 min	n.a.	0.0036	0.0018

Supplementary Figure 7. The lack of chloroplast DNA degradation in biparental zygotes. Changes in relative quantity between nuclear and chloroplastic DNA show that cpDNA mostly survive in biparental zygotes of *otu2p*-ko × *otu2m*-ko and wild-type *plus* × *cOTU2p minus*. gDNA was prepared from the zygotes at the indicated times. Quantitative PCR amplification of two chloroplastic loci (*psbB* and *psbD*) and one nuclear locus (*RCK2*). Relative copy numbers of *psbB* and *psbD* were calculated relative to *RCK2*. Experimental data represent the average ± s.d. from three biological replicates. Letters above the error bars indicate significant differences relative to samples at 30 min (a) or wild-type samples at the same time after mating (b) using student *t*-test (two-tailed, $p < 0.05$). All *p*-values are provided in the table.

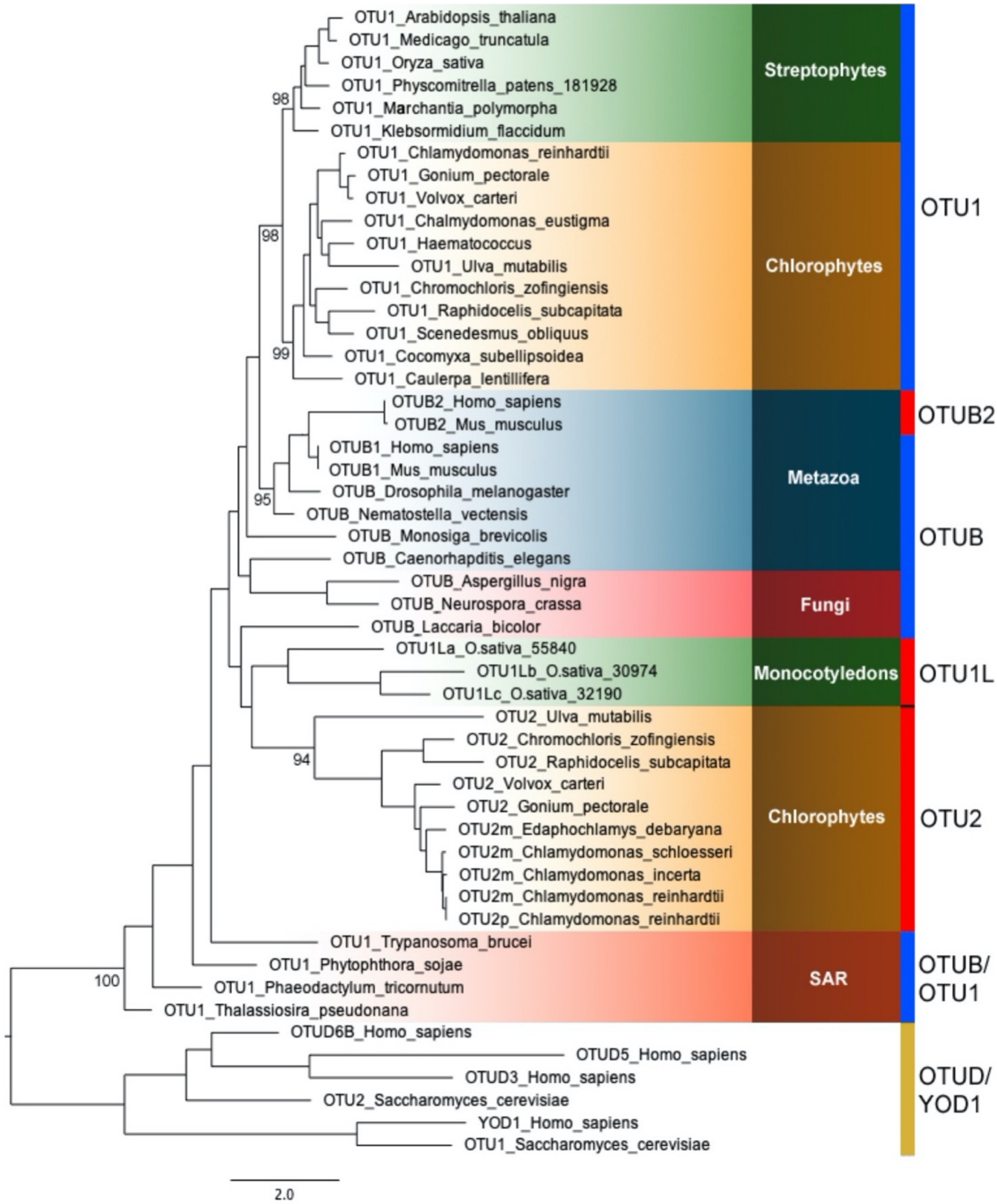

Supplementary Figure 8. Maximum likelihood phylogeny of OTU family DUBs. The HMMsearch identified a single member in the majority of genomes with three exceptions (red bars): mammals (OTUB1 and OTUB2), monocotyledonous plants (OTU1 and OTU1L), and Chlorophyceae, including *C. reinhardtii* (OTU1 and OTU2). Most single members formed a well-supported OTU1 clade for Viridiplantae and an OTUB clade for animals (blue bars). No

OTUB members were found in Rhodophyta and Glaucophyta. Single members in the SAR clade (Stramenopiles, Alveolates, and Rhizaria) and Euglenozoa were divergent. Outside the core-Reinhardtina lineage, *Chromochloris zofingiensis* and *Raphidocelis subcapitata* in the Sphaeropleales clade and *Ulva mutabilis* in the Ulvophyceae clade contained an OTU2 member, suggesting a deeper origin of OTU1/OTU2 diversification in Chlorophytes. The tree was reconstructed using IQ-TREE, based on the LG+I+G4 substitution model, and the bootstrap scores at the nodes were calculated by the Ultrafast method. Selected OTUD family members were included as outgroup sequences (yellow bar).

a

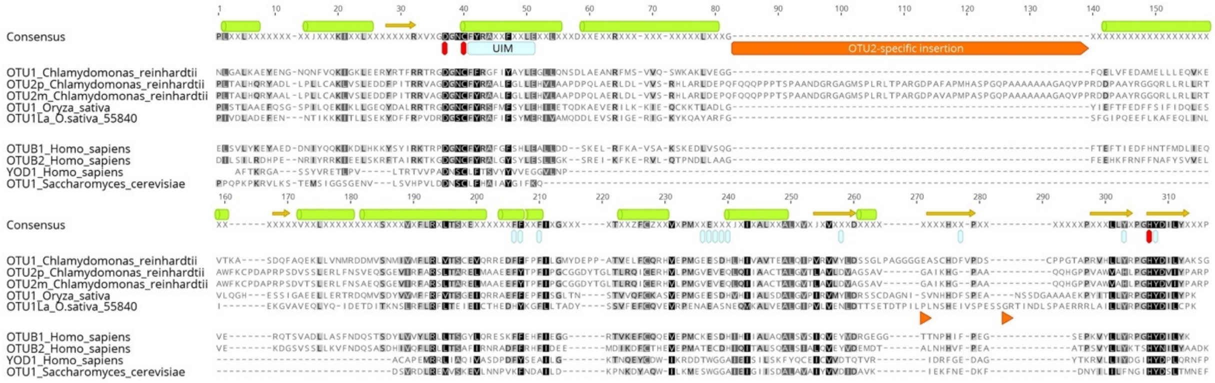

Supplementary Figure 9. Alignment of OTU domain aa sequences of the selected OTUB proteins. (a) Alignment of aa sequences for those depicted in Fig. 2a. OTU domains of OTU1 (aa 49–297Δ6), OTU2 (aa 866–1166), OsOTU1 (aa 33–274), OsOTU1La (aa 239–548Δ65), OTUB1 (human, 53–170 aa), OTUB2 (aa 14–234), YOD1 (human, aa 88–238, outgroup), and ScOTU1 (aa 41–232). Predicted alpha-helices and beta-sheets are indicated above as green rods and yellow arrows, respectively. The catalytic triad residues (D, C, and H) for cys-protease and ubiquitin-interacting residues are highlighted by red and light blue, respectively, below the consensus sequence. (b) Alignment of aa sequences of OTU domains used for the phylogeny in Supplementary Fig. 8 (pages 12–19).

b (1/8)

```
                                       1         10        20        30        40        50        60
                                       |         |         |         |         |         |         |
    OTU2p_Chlamydomonas_reinhardtii   PLTALHQRYADL---LPLLCAKLVSLEDDFPITRRVAGDGNCFYRAALFGLLEHVLAAPD
    OTU2m_Chlamydomonas_reinhardtii   PLTALHQRYAAL---LPLLCAKLVSLEDDFPITRRVAGDGNCFYRAALFGLLEHVLAAPD
        OTU2m_Chlamydomonas_incerta   PLTTLHQRYADL---LPLLCAKLVSLEDDFPATRRVAGDGNCFYRAALFGLLEHVLAAPD
    OTU2m_Chlamydomonas_schloesseri   PLNALHQRYADL---LPLLCAKLVSLEDDFPTTRRVAGDGNCFYRAALFGLLEHVLAAPD
      OTU2m_Edaphochlamys_debaryana   PLQALHQRYAEP---DPLLCAKLVSLEDDFPSVRRVAGDGNCFYRACLFALLEHVLAEPD
                OTU2_Volvox_carteri   PLTVLHQRYAQL---DPLLSAKLVSLEDDFPRTRRVAGDGNCFYRAALFAVLEHVLTVPD
              OTU2_Gonium_pectorale   ------------------------------------------------------------
    OTU2_Chromochloris_zofingiensis   PLGQLIARYEDS---SPLLAAKLLSLEAEFPECRRICGDGNCFYRGFLFGILEALLLEPN
      OTU2_Raphidocelis_subcapitata   PMSRLVERYRLS---SPALAARLAALAADFPTCRAVQGDGNCFYRGFVFALLEALLEQPH
                OTU2_Ulva_mutabilis   PIAHLISTCQH----KPALKAKLEMLLRHFSQYRPILGDGNCFYRSYAFGMIESVLAGWQ
                 OTUB1_Homo_sapiens   ELSVLYKEYAED---DNIYQQKIKDLHKKYSYIRKTRPDGNCFYRAFGFSHLEALLDD--
                OTUB1_Mus_musculus   ELSVLYKEYAED---DNIYQQKIKDLHKKYSYIRKTRPDGNCFYRAFGFSHLEALLDD--
       OTUB_Drosophila_melanogaster   PLTCLYAEYSG----DEIFTAKIQDLSKKYKFIRRTRPDGNCFFRAFAYSYLEYLISN--
        OTUB_Caenorhapditis_elegans   PFSILCAEYDNET--SAAFLSKATELSEVYGEIRYIRGDGNCFYRAILVGLIEIMLKD--
        OTUB_Nematostella_vectensis   -----------------------QSLSAQYSHVRRTRGDGNCFFRAFGFSYFEKLLTD--
                 OTUB2_Homo_sapiens   DILSILRDHPE----NRIYRRKIEELSKRFTAIRKTKGDGNCFYRALGYSYLESLLGK--
                OTUB2_Mus_musculus   DILSILRDHPE----NRIYQRKIQELSKRFTSIRKTKGDGNCFYRALGYSYLESLLGK--
          OTUB_Monosiga_brevicolis   GIDVLLAEYP-----DPLFHTKLETLQRRQASFRRTRGDGNCFYRSYAFGILEHLLQH--
         OTU1_Arabidopsis_thaliana   PLSSLAAEYQSG---SPILLEKIKILDSQYIGIRRTRGDGNCFFRSFMFSYLEHILESQD
          OTU1_Medicago_tranculata   PLSSLAAEYQSG---SPILLEKIKVLDGQYAAIRRTRGDGNCFFRGFMFSYLEHILEAQD
                 OTU1_Oryza_sativa   PLSTLAAEFQSG---SPILQEKIKLLGEQYDALRRTRGDGNCFYRSFMFSYLEHILETQD
             OTU1La_O.sativa_55840   PIVDLADEFEN-----NTIKKKITLLSEKYDFFRPVDRDGSCFYRAFIFSYMERIVAMQD
             OTU1Lb_O.sativa_30974   ANHYFRNIDNSFQ--QPEVGLRLKYLSHHYSEFRPVHRDEECFYRSFIFSYLEQVVDSIG
             OTU1Lc_O.sativa_32190   PLASAPEHYGLRSGLRRIMSANLLILLNEYSDFRP-----------------EQVLDMVG
         OTU1_Marchantia_polymorpha   PLSVLAAEYEAG---NPVFRAKVEKLGQTYVAIRRARGDGNCFFRSFMFSYLEHILETRD
  OTU1_Physcomitrella_patens_181928   PLSVLEEEYKAG---SRVFLAKLQILKQKYGAIRRARGDGNCFFRSFMFSYLEHLLVTQD
        OTU1_Klebsormidium_flaccidum   PLSALAAEYERG---NEVFKAKIQKLGETYGSIRRTRGDGNCFFRGFMFAYLERLLHSQD
        OTU1_Haematococcus_pluvialis   DLSVLKAEYEAG---SDVWVRKIEWLEHKFSHFRRTRGDGNCFYRAFIFSYLEGLLLSQD
     OTU1_Chlamydomonas_reinhardtii   NLGALKAEYENG---NQNFVQKIGKLEERYRTFRRTRGDGNCFFRGFIYAYLEGLLQNSD
              OTU1_Gonium_pectorale   PMSALKEEYING---NQNFVQKIAKLEASYSTFRRTRGDGNCFFRGFIYSYLESLLLARD
                OTU1_Volvox_carteri   PMSALKEEYING---NQNFVQKIGKLEASYGTFRRTRGDGNCFFRGFIFAYLENLLLTHD
        OTU1_Chalmydomonas_eustigma   DMSCLKTEYQNG---NFVFLKKIDSLQATYSSYRRTRGDGNCFYRAFLYRYLESLLDENN
    OTU1_Chromochloris_zofingiensis   PLASLKAEYSAG---SDVFIRKIELLERDFTCMRRTRGDGNCFYRSFIFAYLEWLLATDN
      OTU1_Raphidocelis_subcapitata   TLESLMQDYRA----YPVFVAKIRSLASRYGGIRRVRSDGNCFFRATAFGLLEWLLVNEA
         OTU1_Scenedesmus_obliquus   SLDALKAEYANG---SQVFVAKISNLQTQYSSMRRTRGDGNCFFRSFIYGYLEWLLLHQQ
                OTU1_Ulva_mutabilis   QVLSLREEYQDS---SSTFVAKVEYLCSDYTFIRRTRQDGNCFYRAFLYGLLEALLVTRA
         OTU1_Caulerpa_lentillifera   ------------------------------------------------------------
        OTU1_Cocomyxa_subellipsoidea   PLSALEEEYRQG---SAVFRDKIRSLETQYGSMRRTRGDGNCFFRSFMFAFMEQLVQNND
      OTU1_Thalassiosira_pseudonana   PIDALVAQYSSSN--DGGFLQSANLLSQQYTHLRRIRGDGNCYYRAFLYSLSEDNSKD--
     OTU1_Phaeodactylum_tricornutum   PILDLKLEYNESN--ESRFLDGVDALSKEYQSFRKVRGDGNCYYRAFLYRLVEEIRQN--
            OTU1_Phytophthora_sojae   SPLCLKAEYVGNV--NANFMHGIESLNARYEALRRVRGDGNCFFRGFIFALCERLLPSDS
            OTU1_Trypanosoma_brucei   DGCNLMTEFSD----NPPMLVKVVSLETKYKGIRYARRDGNCFYRSVVFGMHESLLNNKE
              OTU1_Laccaria_bicolor   PMSNLREEYENG---SAAFVNQIDSLTARYDSVRRTRGDGDCFYRSLAFAYVERILQSPE
             OTU1_Aspergillus_nigra   SSSVIAMEYANA---DPAFATKSNALALTHPWTRTMKGDGNCGWRAVAFGYFENLL-LQN
            OTU1_Neurospora_crassa   PSTAITEEYAKA---DPVYVQKTQSLPAVYSHYRPMLGDGNCGWRAIGFGYFETLIKLGN
                 YOD1_Homo_sapiens   ---AFTKRGA-----SSYVRETLPV-----LTRTVVPADNSCLFTSVYYVVEGGVLNP--
      OTU1_Saccharomyces_cerevisiae   PPQPKPKRVLKS---TEMSIGGSGENV---LSVHPVLDDNSCLFHAIAYGIFKQ------
               OTUD6B_Homo_sapiens   EIENLTGARHME---SEKLAQILAARQ---LEIKQIPSDGHCMYKAIEDQLKEKDCA---
      OTU2_Saccharomyces_cerevisiae   ALEASKQPDLKKM--EQESIDQLCELKK--LKQFDIQPDGHCLFASILDQLKLRHDP---
                 OTUD5_Homo_sapiens   IEAMDPATVEQQ---EHWFEKALRDKKG--FIIKQMKEDGACLFRAVADQVYGDQ-----
                 OTUD3_Homo_sapiens   PESGGGGGCEEE---FVSFANQLQALG---LKLREVPGDGNCLFRALGDQLEGHS-----
```

b (2/8)

```
OTU2p_Chlamydomonas_reinhardtii      ---PQLAERLDL-VVS-RHLARLDEPQFQQQP---PPTSP-AANDGRGAGMSPLRLTPAR
OTU2m_Chlamydomonas_reinhardtii      ---PQLAERLDL-VVS-RHLARLDEPQFQQQP---PPTSP-AANDGRGAGMSPLRLTPAR
OTU2m_Chlamydomonas_incerta          ---PKLAERLDL-VVS-RHLARLDEPQFQQQQQQQQHPSPGPASDGRGAGVSPLR--SAR
OTU2m_Chlamydomonas_schloesseri      ---PQLAERLDL-VVS-RHLAQLDEPQFQQQQQQQPGSSP-AAGDARGAGLSPLR--SAR
OTU2m_Edaphochlamys_debaryana        ---PPLGARLEA-AFA-AHLRTLDEPPAASAT---TAAA--APAAGSAGGATSPR--TPR
OTU2_Volvox_carteri                  AV-LPNRSRLHELRLE-HLVGRYVRKLEALQDVGGVPQG--GGDEGAGGGRSRSR--AAT
OTU2_Gonium_pectorale                ---PSAPYSLPGDSLP-GSARREAVRPE-------PAP---GAASAASGSRSAAR-----
OTU2_Chromochloris_zofingiensis      ---LTLYARLRA-SIE-HTWLEMRAHM---------------------------------
OTU2_Raphidocelis_subcapitata        ---VELHARLLQRVQEAWQLARTRLPP---------------------------------
OTU2_Ulva_mutabilis                  RF-KLKSCTVAA-QTW-VWKAQALDIP---------------------------------
OTUB1_Homo_sapiens                   ---SKEL-RFKA-VSA-KSKEDLVSQG---------------------------------
OTUB1_Mus_musculus                   ---SKELQRFKA-VSA-KSKEDLVSQG---------------------------------
OTUB_Drosophila_melanogaster         ---TSAY-EFKK-LAE-ESKEKLVQLG---------------------------------
OTUB_Caenorhapditis_elegans          ---RARLEKFIA-SSR-DWTRTLVELG---------------------------------
OTUB_Nematostella_vectensis          ---EKEYQRFKD-LAA-RSKDELVELN---------------------------------
OTUB2_Homo_sapiens                   ---SREI-KFKE-RVL-QTPNDLLAAG---------------------------------
OTUB2_Mus_musculus                   ---SREI-KFKE-RVL-QTPNDLLAAG---------------------------------
OTUB_Monosiga_brevicolis             ---KDAIDGFLL-RLR-ETYEMALAAG---------------------------------
OTU1_Arabidopsis_thaliana            ---RAEVDRIKV-NVE-KCRKTLQNLG---------------------------------
OTU1_Medicago_tranculata             ---SAEIDRIKA-NVE-RSRKALQTLG---------------------------------
OTU1_Oryza_sativa                    ---KAEVERILK-KIE-QCKKTLADLG---------------------------------
OTU1La_O.sativa_55840                ---DLEVSRIGE-RIG-KYKQAYARFG---------------------------------
OTU1Lb_O.sativa_30974                ---TREEDRLLA-AVR-ALATKAENLQ---------------------------------
OTU1Lc_O.sativa_32190                ---TDEENRLLA-AVG-AIDHRQ-------------------------------------
OTU1_Marchantia_polymorpha           ---ETEVKRIQK-NIE-QSKKALIELG---------------------------------
OTU1_Physcomitrella_patens_181928    ---VAEVARMEK-AIE-ICKKTLIDHG---------------------------------
OTU1_Klebsormidium_flaccidum         ---KTELPRMRD-RIE-RCKQELIAIG---------------------------------
OTU1_Haematococcus_pluvialis         ---TQETKRLAE-VLQ-GWRSKLVEAG---------------------------------
OTU1_Chlamydomonas_reinhardtii       ---LAEANRFMS-VVQ-SWKAKLVEGG---------------------------------
OTU1_Gonium_pectorale                ---LTECDRIKS-VIQ-EWKGKLVDGG---------------------------------
OTU1_Volvox_carteri                  ---LPECSRFMS-VVQ-AWKTRLVEGG---------------------------------
OTU1_Chalmydomonas_eustigma          ---IPECRRFIE-CIN-GWRQKLVEAH---------------------------------
OTU1_Chromochloris_zofingiensis      ---EQQCQRFSN-CVG-GWRQKLIDAG---------------------------------
OTU1_Raphidocelis_subcapitata        ---DSECQNFLR-TLE-ACKDRMVGAG---------------------------------
OTU1_Scenedesmus_obliquus            ---EQECQRFTK-CLQ-GWKQKLQDIG---------------------------------
OTU1_Ulva_mutabilis                  ---VEATQAMLR-MMH-PLRQMLLDAG---------------------------------
OTU1_Caulerpa_lentillifera           ---QQNQLRMKD-HME-TCKKKLLDAG---------------------------------
OTU1_Cocomyxa_subellipsoidea         ---LSERNRVVT-CIR-QWKAKLVGVG---------------------------------
OTU1_Thalassiosira_pseudonana        ---DGEFERIKQ-LVS-DSLLWVCKFG---------------------------------
OTU1_Phaeodactylum_tricornutum       ---EKDSEKIID-WLKTKSWESVLAAG---------------------------------
OTU1_Phytophthora_sojae              GG-AALRGRIQH-KIQ-QSKSELVAIG---------------------------------
OTU1_Trypanosoma_brucei              RA-GAHLERITD-----LSRQVVAD-----------------------------------
OTU1_Laccaria_bicolor                P--DFAVASSVS-ALA-ATRDSLDKAG---------------------------------
OTU1_Aspergillus_nigra               ---LMQVRHELA-RIK-SMNALLDQTG---------------------------------
OTU1_Neurospora_crassa               ---KAQVDGERE-RLE-SLNEYIQHHG---------------------------------
YOD1_Homo_sapiens                    ------------------------------------------------------------
OTU1_Saccharomyces_cerevisiae        ------------------------------------------------------------
OTUD6B_Homo_sapiens                  ------------------------------------------------------------
OTU2_Saccharomyces_cerevisiae        ------------------------------------------------------------
OTUD5_Homo_sapiens                   ------------------------------------------------------------
OTUD3_Homo_sapiens                   ------------------------------------------------------------
```

b (3/8)

```
OTU2p_Chlamydomonas_reinhardtii     GDPAFAPMHASPGQPAAAAAAAAGAQVPPRDDPAAYRGGQRLLRLLRTAWFKCPDA-----
OTU2m_Chlamydomonas_reinhardtii     GDPAVAPMPASPGQPAAAAAAAAGAQVPPRDDPAAYRGGQRLLRLLRTAWFKCPDA-----
OTU2m_Chlamydomonas_incerta         GDPAGTLAPASPGQPGEGAAA--AHIPPRDDPAAYRGGQRLLRLLRTAWFKCPDA-----
OTU2m_Chlamydomonas_schloesseri     GDTVGS-TPASPSPRRESTAA--AQVPPRDDPAAYRGGQRLLRLLRTAWFKCPDA-----
OTU2m_Edaphochlamys_debaryana       T------SPRAAGNGGTASAD--GPLPPSQDAAAYNGGRLLLRLLRRAWFKAPDA-----
OTU2_Volvox_carteri                 PE-----RGGRSDSGGGGGGG--GGGGRSVDQVALRGGKRLLRLLRTAWFKAPDA-----
OTU2_Gonium_pectorale               -----------------------PSSSSSSATRLRQRSEPDAAGARAAVPRGPDT-----
OTU2_Chromochloris_zofingiensis     ----------------------------QVDADTERGFNVLANFLSRTWYVPAAQHADSR
OTU2_Raphidocelis_subcapitata       ---------------------------NPEVERGAALLTRLLARVWYAPGAAGSVEAAAA
OTU2_Ulva_mutabilis                 ----------------------GHASFKARSDKLLKDGYVKFRELKDQVERA--------
OTUB1_Homo_sapiens                  ----------------------------FTEFTIEDFHNTFMDLIEQVE-----------
OTUB1_Mus_musculus                  ----------------------------FTEFTIEDFHNTFMDLIEQVE-----------
OTUB_Drosophila_melanogaster        ----------------------------FPSFTLEDFHETFMEVIQRVSPDNAG------
OTUB_Caenorhapditis_elegans         ----------------------------FPDWTCTDFCDFFIEFLEKIH-----------
OTUB_Nematostella_vectensis         ----------------------------FPAFTIEDFHEVFMEVLDMMG-----------
OTUB2_Homo_sapiens                  ----------------------------FEEHKFRNFFNAFYSVVELVE-----------
OTUB2_Mus_musculus                  ----------------------------FEEHKFRNFFNAFYSVVELVE-----------
OTUB_Monosiga_brevicolis            ----------------------------CPEFTTEDFYETVADMVTDLK-----------
OTU1_Arabidopsis_thaliana           ----------------------------YTDFTFEDFFALFLEQLDDILQGT--------
OTU1_Medicago_tranculata            ----------------------------YAELTFEDFFTLFLEQLEDVIQGK--------
OTU1_Oryza_sativa                   ----------------------------YIEFTFEDFFSIFIDQLESVLQGH--------
OTU1La_O.sativa_55840               ----------------------------SFGIPQEEFLKAFEQLINLI------------
OTU1Lb_O.sativa_30974               ----------------------------WASE-FSQKHKAFERLIEKIKGWKRMQEH---
OTU1Lc_O.sativa_32190               ----------------------------WASG-FSQSHKVFEKLIQNVMRWKRRQKGVA-
OTU1_Marchantia_polymorpha          ----------------------------YQEFTFEDFLSIFVEQLDSVLPGR--------
OTU1_Physcomitrella_patens_181928   ----------------------------FAEFTFEDFLAIFVEQLQSVIQDK--------
OTU1_Klebsormidium_flaccidum        ----------------------------YTPFTFEDFLEIFMDQLESVLPEE--------
OTU1_Haematococcus_pluvialis        ----------------------------FQDLVFSDALDLLLEQLNSIS-----------
OTU1_Chlamydomonas_reinhardtii      ----------------------------FQELVFEDAMELLLEQVKEVTKA---------
OTU1_Gonium_pectorale               ----------------------------FQALVFEDAMDVLLEQVAEISKP---------
OTU1_Volvox_carteri                 ----------------------------FQELVFEDAMDVLLDQVKEITKP---------
OTU1_Chalmydomonas_eustigma         ----------------------------FQELVFEDAMELLLEQVSTIGNN---------
OTU1_Chromochloris_zofingiensis     ----------------------------FQELVFEDAMDILQNMVNSISLGPQV------
OTU1_Raphidocelis_subcapitata       ----------------------------FDPLVFEDALDMLAGMVNAICVGASVP-----
OTU1_Scenedesmus_obliquus           ----------------------------YDSIVFEDPLDMLLQQVQAILIGPGV------
OTU1_Ulva_mutabilis                 ----------------------------FMELVFEHAMEVLEDLLKAVANT---------
OTU1_Caulerpa_lentillifera          ----------------------------FQELVFEDALAILQQRVEQIGT----------
OTU1_Cocomyxa_subellipsoidea        ----------------------------FQEMVFEDAMEIVIDQLNSLGT----------
OTU1_Thalassiosira_pseudonana       ----------------------------YDEITVDMFYEELVDLLEFIESH---------
OTU1_Phaeodactylum_tricornutum      ----------------------------YDEVMLEAFHDTIVELLERILA----------
OTU1_Phytophthora_sojae             ----------------------------YSDVAIDAFWETFVDYLAAME-----------
OTU1_Trypanosoma_brucei             ----------------------------YGNF-AEDFCHPAVEMAKKIES----------
OTU1_Laccaria_bicolor               ----------------------------IDKIVYEDIYDEFVGLISTIVQPNSA------
OTU1_Aspergillus_nigra              ----------------------------QQEHLYEIFVDATEDVFIQIATAIQ-------
OTU1_Neurospora_crassa              ---------------------------GFDPMVFVDFVEETDELLKRISDKLN-------
YOD1_Homo_sapiens                   ------------------------------------------------------------
OTU1_Saccharomyces_cerevisiae       ------------------------------------------------------------
OTUD6B_Homo_sapiens                 ------------------------------------------------------------
OTU2_Saccharomyces_cerevisiae       ------------------------------------------------------------
OTUD5_Homo_sapiens                  ------------------------------------------------------------
OTUD3_Homo_sapiens                  ------------------------------------------------------------
```

b (4/8)

```
OTU2p_Chlamydomonas_reinhardtii      -----------PRPSDVSSLERLFNSVEQSGEVIRFARSLTARELMAAEEFYTPFIPGCG
OTU2m_Chlamydomonas_reinhardtii      -----------PRPSDVTSLERLFNSAEQSGEVIRFARSLTARELMAAEEFYTPFIPGCG
OTU2m_Chlamydomonas_incerta          -----------PRPSDVSSLERLFNSAEQSGEVIRFARSLTARELMAAEDFYTPFIPGCG
OTU2m_Chlamydomonas_schloesseri      -----------PRPSDVSSLERLLNSAEQSGEVIRFARSLTARELMAAEEFYTPFIPGCG
OTU2m_Edaphochlamys_debaryana        -----------PAPTDLASLEALLNAPGDSGDVIRFARSLTVRELLRAEDFYAPFIPGCG
OTU2_Volvox_carteri                  -----------PEPSDVPSRKRLFNSSAASSEVIRFARSLTVYELVSDEAFYAPFIPGCG
OTU2_Gonium_pectorale                -----------DSPSDLSSLERLLNSGSASAEVIRFARSLTVAELMAGEEFYAPYIPGCG
OTU2_Chromochloris_zofingiensis      LPAAAQQEEG-NGPLYLPELEAALSSRDIFDPLVKFARSATSWELKSNEQMYAPFIAGCC
OTU2_Raphidocelis_subcapitata        ARAGAGASTG-AGPLFLPELEAALACRDAFDPLVAFARAATSLELRANEAAYAPFLAGCG
OTU2_Ulva_mutabilis                  -----------GENVDPMPLVLEFFQGVMGNSVMHLIRQLAAYEMLAHEDQYATFLPAIE
OTUB1_Homo_sapiens                   ------------RQTSVADLLASFNDQSTSDYLVVYLRLLTSGYLQRESKFFEHFIEGG-
OTUB1_Mus_musculus                   ------------KQTSVADLLASFNDQSTSDYLVVYLRLLTSGYLQRESKFFEHFIEGG-
OTUB_Drosophila_melanogaster         -----------GHSTVQDELHKIFNEQGYSDYVVVYLRLITSGKLQEEADFYQNFIEGD-
OTUB_Caenorhapditis_elegans          -----------SGVHTEEAVYTILNDDGSANYILMFFRLITSAFLKQNSEEYAPFIDEG-
OTUB_Nematostella_vectensis          ------------NNATEDQLIQKFQDEGISNYLVVYLRLLTSAQLQRNAVFFENFIEGE-
OTUB2_Homo_sapiens                   ------------KDGSVSSLLKVFNDQSASDHIVQFLRLLTSAFIRNRADFFRHFIDEE-
OTUB2_Mus_musculus                   ------------KDSSVSSLLKVFNDQSSSDRIVQFLRLLTSAFIRNRADFFRHFIDEE-
OTUB_Monosiga_brevicolis             -----------ADEASLTTLLAAVNDEGLSNYLVAYMRIITSAYMQLNADYFQPFIEGG-
OTU1_Arabidopsis_thaliana            -----------EESISYDELVNRSRDQSVSDYIVMFFRFVTAGDIRTRADFFEPFITGLS
OTU1_Medicago_tranculata             -----------ETSISHEELVLRSRDQSVSDYVVMFFRFVTSAEIQKRSEFFEPFIMGLT
OTU1_Oryza_sativa                    -----------ESSIGAEELLERTRDQMVSDYVVMFFRFVTSGEIQRRAEFFEPFISGLT
OTU1La_O.sativa_55840                -----------EKGVAVEQLYQ-IDETDITKNSLRFLRFLTEIEICTHEDHYKGFLLTAD
OTU1Lb_O.sativa_30974                -----------PISIIRGKILEFFSSYDTTDDIFAFLRLVAATWMCTRIWNYEWCATNCG
OTU1Lc_O.sativa_32190                -----------SADSRRQKLLEFFSSYSKSDGILAFLKYAAANWICSHREEYEPNIAGLG
OTU1_Marchantia_polymorpha           -----------ESSVDIDQLVEKCRDRYISDYVVMFFRFVTSAEIRKRAEFFEPFILGLS
OTU1_Physcomitrella_patens_181928    -----------EVSVSLETLVERCRDQYISNSVVMFFRFVTSGEIGRRTEFFEPFIQGMS
OTU1_Klebsormidium_flaccidum         -----------GQPISLSELEVRCRDPNVSNWAVMFFRFVTSAEVQRRAEFFEPFILGLT
OTU1_Haematococcus_pluvialis         -----------QGFLSSEALQEAYQDDTASNLVIMLLRFIVSAEIKRRDDFFLPFILGMY
OTU1_Chlamydomonas_reinhardtii       -----------SDQFAQEKLLVNMRDDMVSNMIVMFLRLVTSCEVQRREDFFFPFILGMY
OTU1_Gonium_pectorale                -----------YTNFAHDKLLVDMRDDTISNLIVMLLRLITSCEVQRREDHFFPFILGMY
OTU1_Volvox_carteri                  -----------YTNYMHERLLVNMRDDTVSNLVVMLLRLITSCEVQRREDFFFPFIMGMY
OTU1_Chalmydomonas_eustigma          -----------DNPMTKETLTINMRDDMVSNMMVMFLRMVTSAEIQRRSDFFAPFIMGMY
OTU1_Chromochloris_zofingiensis      -----------PDHMTQEQLENNFRDDMLSNTVVMFLRMVTSAELQRRQDFFLPFIWGMT
OTU1_Raphidocelis_subcapitata        -----------GQGADASTLLANFRDDTVSNMVVMLLRFITSAEIQARQDHFAPFVLGVA
OTU1_Scenedesmus_obliquus            -----------EGGIDEQQLLANFRDDTWSNTCVMLLRFITSAEIKRRVDHFEPFVVGQY
OTU1_Ulva_mutabilis                  -----------EDRMEPGELLVRLNDEDVCNMLVMLVRFVASAHVSRRAEHFLPFILGMV
OTU1_Caulerpa_lentillifera           -----------ELTLEEWELVMN--DDNMSNYIVMFLRLLTSAEIQLRREFFEPFIMGIS
OTU1_Cocomyxa_subellipsoidea         -----------AEPLSVPQLEDNFCDAMLSPMIIMLLRMLTSAEIQRRSDFFAPFIMGMC
OTU1_Thalassiosira_pseudonana        -----------ASSQLHSKLNE---ENATSDYCTWFMRVMTAAQMKSNPDRYLPFLFGGM
OTU1_Phaeodactylum_tricornutum       -----------GTLDEAAFHEEMNQETATSDYCTWYLRVVTATHLKQDPGRFLPFIAEPG
OTU1_Phytophthora_sojae              ------------TRTHAELVQDFQTEGGESEYLVWYMRLLTAGYMKQHAETFQPFIDGLY
OTU1_Trypanosoma_brucei              -----------GECSTVEQLYELS-TNGEAEYALYFYRYAVSHHIRTHEDDFLPFVVGMG
OTU1_Laccaria_bicolor                -----------GKKLDSAGLLLAFQSPEMSNAVVVYMRFLTSAEIRVHRENYEAFLIHPE
OTU1_Aspergillus_nigra               -----------SGIRDETFLVDAFNEEYNSNAIITHFRLLTSAWMKLHPAQYEAFLSM--
OTU1_Neurospora_crassa               -----------VPEEAMSEVMHAFTDAEKSNAIIYHLRLLASSWLKENRGSHEAFITTD-
YOD1_Homo_sapiens                    -------------------------------ACAPEMRRLIAQIVASDPDFYSEAILG--
OTU1_Saccharomyces_cerevisiae        -------------------------------DSVRDLREMVSKEVLNNPVKFNDAILD--
OTUD6B_Homo_sapiens                  -------------------------------LTVVALRSQTAEYMQSHVEDFLPFLTNPN
OTU2_Saccharomyces_cerevisiae        -------------------------KKLDQDMDVMKLRWLSCNYVQEHRDDFIPYLFDEE
OTUD5_Homo_sapiens                   -------------------------------DMHEVVRKHCMDYLMKNADYFSNYVTE--
OTUD3_Homo_sapiens                   -------------------------------RNHLKHRQETVDYMIKQREDFEPFVEDD-
```

b (5/8)

```
OTU2p_Chlamydomonas_reinhardtii   GDYTGLTLRQICERHVLPMGVEVEQLQIIAACTALGVTLAVLDVAGSAV-----------
OTU2m_Chlamydomonas_reinhardtii   GDYTGLTLRQICERHVLPMGVEVEQLQIIAACTALGVTLAVLDVAGSAV-----------
OTU2m_Chlamydomonas_incerta       GDYAGLSLRQICERHVLPMGVEVEQLQIIAACTALGVTLAVLDVAGSAV-----------
OTU2m_Chlamydomonas_schloesseri   GDYTGLSLRQICERHVLPMGVEVEQLQIIAACTALGVTLAVLDVAGSAV-----------
OTU2m_Edaphochlamys_debaryana     RDYTGLSLRQICAQHVLPLGVEVEQLQMIALCTALGAVVAVLDVAGSQV-----------
OTU2_Volvox_carteri               MDYSGMSLQWICSHHVLPMGVEVEQLQIIALCRALGITLGVLDVAGSQV-----------
OTU2_Gonium_pectorale             GDYSGLSLRQVCVRHVLPMGVEVEQLQINALCSALGAPVAVLDVAGSQV-----------
OTU2_Chromochloris_zofingiensis   QEYTALNIQQICERYVERMGQEVEQLQMVALAHAMKVAVGILDVAGSEI-----------
OTU2_Raphidocelis_subcapitata     PGYGAASMAELCSRRVERMGEEVEQVQMAALASAVAVSVGVLDVAGSEV-----------
OTU2_Ulva_mutabilis               GPYEQLTVPLVVGRLVVADGVEAEHVMIQAIATLLGLCLAVVYADGQA------------
OTUB1_Homo_sapiens                -----RTVKEFCQQEVEPMCKESDHIHIIALAQALSVSIQVEYMDRGEGG----------
OTUB1_Mus_musculus                -----RTVKEFCQQEVEPMCKESDHIHIIALAQALSVSIQVEYMDRGEGG----------
OTUB_Drosophila_melanogaster      -----LTIEAFRHLEVEPMYKESDHIHIIALCTALGAGVRVEYLDRGEGG----------
OTUB_Caenorhapditis_elegans       -----MTVAQYCEQEIEPMWKDADHLAINSLIKAAGTRVRIEYMDRTAAP----------
OTUB_Nematostella_vectensis       -----RTVKEFCNQEVEPMGKESDHIHIIGLTEALGVCIRVEYVDRTGD-----------
OTUB2_Homo_sapiens                -----MDIKDFCTHEVEPMATECDHIQITALSQALSIALQVEYVDEMDT-----------
OTUB2_Mus_musculus                -----MDIKDFCTHEVEPMAMECDHVQITALSQALNIALQVEYVDEMDT-----------
OTUB_Monosiga_brevicolis          -----LTVKEYCSISVDPFGVESEHLQAQALISALRLGCRITYIDRSPGA----------
OTU1_Arabidopsis_thaliana         N----ATVDQFCKSSVEPMGEESDHIHITALSDALGVAIRVVYLDRSSCDSGGV------
OTU1_Medicago_tranculata          N----TTVEQFCKSAVEPMGEESDHVHITALSDALGIPIRVVYLDRSSCDTGGV------
OTU1_Oryza_sativa                 N----STVVQFCKASVEPMGEESDHVHIIALSDALGVPIRVMYLDRSSCDAGNI------
OTU1La_O.sativa_55840             Y----SSVFEFCQVEVRPENAEASNEQMKALVEALGIPVLVENLDTTSETDTP-------
OTU1Lb_O.sativa_30974             EN---QNLEDWCSKHVIAPRVYATSAAVKACAEALRVTVQVENVHDGT------------
OTU1Lc_O.sativa_32190             GG---YTLEAWCEIYLLQPREQTDHIQMTAVAAALGVPLRVENLHNGP------------
OTU1_Marchantia_polymorpha        N----MNVQQFCKTSVEPMGEESDHVHITALSDALGVPVRVVYLDRSVGADGKNT-----
OTU1_Physcomitrella_patens_181928 N----MSVVQFRRSSVEPMGEESDHVHITALSDALGVPVRVVYLDQSGDVNDKPV-----
OTU1_Klebsormidium_flaccidum      N----LDVKKFCQTSVEPMGEESDHVHITALTDALGIPVRIVYLDRSGGASDDAL-----
OTU1_Haematococcus_pluvialis      DDPP-ACVDTFCQRYVEPMAEESDNIHIVALTDALQVPVTVVYLDRSGVESRASHLATRT
OTU1_Chlamydomonas_reinhardtii    DEPP-ATVELFCQRHVEPMGEESDHLHIVAVTEALQIPVRVVYLDSSGLPAGGGGGGAGA
OTU1_Gonium_pectorale             DEPP-ATVELFCQRHVEPMGEESDHLHIVAITDALQIPVRVVYLDSSGLPTGGGSGL---
OTU1_Volvox_carteri               DEPP-ATVELFCQRHVEPMGEESDHLHIVAITEALQIPVRVVYLDSSGMPTGGNGSGL--
OTU1_Chalmydomonas_eustigma       DDV--SCVEVFCQRSVEVMGEESDHVHIVALSDALQVPIAVVYLDRSNVNRVNEGSSEV-
OTU1_Chromochloris_zofingiensis   EDES-MTADVFCRKFVEPMGEESEHIHAQVLTDAFQVAVRVMYLDSRDHGTDN-------
OTU1_Raphidocelis_subcapitata     D----TDVQTFCRRFVEPMAEESDHVHAQALCDALRVPITVVYLDSHERSGVGGGAGGST
OTU1_Scenedesmus_obliquus         D----MDVATFCERSVDVMGEESDHIHAQALTDAVQVPVRIIQIDSSGGQ----------
OTU1_Ulva_mutabilis               DGC--DGVDDFRRRHVEAMGEESDHIQVVAVTEAFQVPVLVVYLDNSSVTADGNGNGNGN
OTU1_Caulerpa_lentillifera        DV---LDVELFCQRMVEVMDEESDEIHIRALTDALEIPTRVYSLDSQPPI----------
OTU1_Cocomyxa_subellipsoidea      DDE--VSVEQFCRRYVEPMGEESDHVHIVALTNALLVPIRVVYLDRSMGAAMAGVGYDSA
OTU1_Thalassiosira_pseudonana     -----IDIPTFCTREVEPMNKECGMVQVAALAECFGVRVAIEYMDGRR------------
OTU1_Phaeodactylum_tricornutum    -----LGINDFCQREVEPMGKECEQVQVLALAEAFGVQVTIAYLDGHELLYG--------
OTU1_Phytophthora_sojae           PG---QTVAQFCAAEVEPMGKECDQPQIAALTEALQVGVKIEYLDGSAGPGQ--------
OTU1_Trypanosoma_brucei           H----ETVSEFCSVEVDAVASEADNVQVVAFAQCFDVRVIVEYVDGREGD----------
OTU1_Laccaria_bicolor             THEP-MGVDAFCANIVEPLGKEADHVEIQALCAALQLNVDVAYLNGGSEE----------
OTU1_Aspergillus_nigra            ------PVEQYCATRIDVVRTEIDEIGLQALVDGVGFAVEIMYLDRSEGE----------
OTU1_Neurospora_crassa            -----ESVEKFCQRVIEVPNIEIDQLGLQALVSILGFVLRVAYLDRSPGS----------
YOD1_Homo_sapiens                 -----KTNQEYCDW-IKRDDTWGGAIEISILSKFYQCEICVVDTQTVR------------
OTU1_Saccharomyces_cerevisiae     -----KPNKDYAQW-ILKMESWGGAIEIGIISDALAVAIYVVDIDAVK------------
OTUD6B_Homo_sapiens               TGYTPEEFQKYCED-IVNTAAWGGQLELRALSHILQTPIEIIQADSPP------------
OTU2_Saccharomyces_cerevisiae     TMKM-KDIDEYTKE-MEHTAQWGGEIEILALSHVFDCPISILMSGRP-------------
OTUD5_Homo_sapiens                ------DFTTYINR-KRKNNCHGNHIEMQAMAEMYNRPVEVYQYSTEP------------
OTUD3_Homo_sapiens                -----IPFEKHVAS-LAKPGTFAGNDAIVAFARNHQLNVVIHQLNAPLW-----------
```

b (6/8)

```
OTU2p_Chlamydomonas_reinhardtii  GAIKHG--PAA-------------------------------------------------
OTU2m_Chlamydomonas_reinhardtii  GAIKHG--PAA-------------------------------------------------
OTU2m_Chlamydomonas_incerta  GAIKHG--PAA-------------------------------------------------
OTU2m_Chlamydomonas_schloesseri  GAIKHG--PAA-------------------------------------------------
OTU2m_Edaphochlamys_debaryana  GAIKHG--PPG-------------------------------------------------
OTU2_Volvox_carteri  GAIKHG--PTG-------------------------------------------------
OTU2_Gonium_pectorale  GAIKHG--PPG-------------------------------------------------
OTU2_Chromochloris_zofingiensis  GYIQH---PAG-------------------------------------------------
OTU2_Raphidocelis_subcapitata  GMVRH---PAG-------------------------------------------------
OTU2_Ulva_mutabilis  DPKVHIISSQR-------------------------------------------------
OTUB1_Homo_sapiens  TTNPHIF-PEG-------------------------------------------------
OTUB1_Mus_musculus  TTNPHVF-PEG-------------------------------------------------
OTUB_Drosophila_melanogaster  TVKAHDF-PEG-------------------------------------------------
OTUB_Caenorhapditis_elegans  NGGWHYDIPSD-------------------------------------------------
OTUB_Nematostella_vectensis  DVNHHDF-PED-------------------------------------------------
OTUB2_Homo_sapiens  ALNHHVF-PEA-------------------------------------------------
OTUB2_Mus_musculus  ALNHHVF-PEA-------------------------------------------------
OTUB_Monosiga_brevicolis  EPPCHVLEGPD-------------------------------------------------
OTU1_Arabidopsis_thaliana  TVNHHDFVPVG-------------------------------------------------
OTU1_Medicago_tranculata  SVNHHDFTPVA-------------------------------------------------
OTU1_Oryza_sativa  SVNHHDFSPEA-------------------------------------------------
OTU1La_O.sativa_55840  ILNQHFIYPRPESEEGTMLGPLNSHEIVSPESSGYHAARGELQNQPSTSGSSTNSSTEAL
OTU1Lb_O.sativa_30974  CESTHYIVRGA-------------------------------------------------
OTU1Lc_O.sativa_32190  AQDIYTADGVN-------------------------------------------------
OTU1_Marchantia_polymorpha  DVNHHDFLPPD-------------------------------------------------
OTU1_Physcomitrella_patens_181928  IVNNHDFIPEG-------------------------------------------------
OTU1_Klebsormidium_flaccidum  EINHHDFVPES-------------------------------------------------
OTU1_Haematococcus_pluvialis  EVNEHTIVPDA-------------------------------------------------
OTU1_Chlamydomonas_reinhardtii  EASCHDFVPDS-------------------------------------------------
OTU1_Gonium_pectorale  DATVHDFVPDS-------------------------------------------------
OTU1_Volvox_carteri  DATMHDFVPDS-------------------------------------------------
OTU1_Chalmydomonas_eustigma  EVETYHFNPEP-------------------------------------------------
OTU1_Chromochloris_zofingiensis  SVSKVDFIPEG-------------------------------------------------
OTU1_Raphidocelis_subcapitata  ECEVHRFEPSD-------------------------------------------------
OTU1_Scenedesmus_obliquus  EASVVDMKPEG-------------------------------------------------
OTU1_Ulva_mutabilis  QCNTHVFEPRE-------------------------------------------------
OTU1_Caulerpa_lentillifera  EIQPTDYVPEE-------------------------------------------------
OTU1_Cocomyxa_subellipsoidea  AVNHHDFVPDS-------------------------------------------------
OTU1_Thalassiosira_pseudonana  -VATHVFGESE-------------------------------------------------
OTU1_Phaeodactylum_tricornutum  RLAQHTFGPDS-------------------------------------------------
OTU1_Phytophthora_sojae  DLQSYVCSPTV-------------------------------------------------
OTU1_Trypanosoma_brucei  CTTRHTFQQKD-------------------------------------------------
OTU1_Laccaria_bicolor  GVVDFIPFRYA-------------------------------------------------
OTU1_Aspergillus_nigra  AVTPHLL-TPP-------------------------------------------------
OTU1_Neurospora_crassa  EVNTYSF-PEE-------------------------------------------------
YOD1_Homo_sapiens  -IDRFGE-DAG-------------------------------------------------
OTU1_Saccharomyces_cerevisiae  -IEKFNE-DKF-------------------------------------------------
OTUD6B_Homo_sapiens  -IIVGEE-YSK-------------------------------------------------
OTU2_Saccharomyces_cerevisiae  -IQVYNE-CGK-------------------------------------------------
OTUD5_Homo_sapiens  -INTFHG-IHN-------------------------------------------------
OTUD3_Homo_sapiens  -QIRGTE-KSS-------------------------------------------------
```

b (7/8)

```
OTU2p_Chlamydomonas_reinhardtii      --------------------------QQHGPPVAWVAHL------PGHYDVIYPARPLDV
OTU2m_Chlamydomonas_reinhardtii      --------------------------QQHGPPVAWVAHL------PGHYDVIYPARPLDV
OTU2m_Chlamydomonas_incerta          --------------------------QQHGPPVAWVAHL------PGHYDVIYPARALDV
OTU2m_Chlamydomonas_schloesseri      --------------------------QQHSPPVAWVAHL------PGHYDVIYPARPLDV
OTU2m_Edaphochlamys_debaryana        -------------------------PAAAQPPAAWVAHL------PGHYDVIYPARRMDV
OTU2_Volvox_carteri                  ---------------------------HLGPPVAWVAHL------PGHYDVVYPAKQLDV
OTU2_Gonium_pectorale                ---------------------------ARGPPAAWVAHL------PGHYDVIYPARPLDV
OTU2_Chromochloris_zofingiensis      ----------------------------DATLAFWMIHL------PGHYEIVYARPGLDV
OTU2_Raphidocelis_subcapitata        ----------------------------GGEPLFYLVHL------PGHYEICYRS-----
OTU2_Ulva_mutabilis                  ------------------------EHVEEGEPSMFLLFR------DGHYDLIYPINNSWR
OTUB1_Homo_sapiens                   -----------------------------SEPKVYLLYR------PGHYDILYK------
OTUB1_Mus_musculus                   -----------------------------SEPKVYLLYR------PGHYDILYK------
OTUB_Drosophila_melanogaster         -----------------------------SEPRIYLIYR------PGHYDILYPN-----
OTUB_Caenorhapditis_elegans          --------------------------DQQIAPEITLLYR------PGHYDVIYKKDSTEA
OTUB_Nematostella_vectensis          ----------------------------GSTPIVHLLYR------PGHYDVLYLANQ---
OTUB2_Homo_sapiens                   -----------------------------ATPSVYLLYK------TSHYNILYAADKH--
OTUB2_Mus_musculus                   -----------------------------AIPSVYLLYK------TSHYNILYAAEKH--
OTUB_Monosiga_brevicolis             ---------------------------GSGQPVCHLMFR------PGHYDVLYLPTAAP-
OTU1_Arabidopsis_thaliana            --------------------ITNEKDEEASAPFITLLYR------PGHYDILYPKPSCKV
OTU1_Medicago_tranculata             -----------------GDLPSASGSSEKKNPFITLLYR------PGHYDILYPK-----
OTU1_Oryza_sativa                    --------------------NSSDGAAAAEKPYITLLYR------PGHYDILYPK-----
OTU1La_O.sativa_55840                GLQSIGTSSTPNERDGKGDRTINDLSPAERRRLAILLYR------PGHYDILCPK-----
OTU1Lb_O.sativa_30974                -------------------------------PCVTLLRI------ESHYDIIYPLPPSSI
OTU1Lc_O.sativa_32190                ------------------------------IPRVTLLYT------GVHYDILYPRHPSGG
OTU1_Marchantia_polymorpha           -------------------------CPTVEMPPVVLLYR------PGHYDILYTREVQNL
OTU1_Physcomitrella_patens_181928    -------------------------MDAAVEPNVILLYR------PGHYDILYRKENSED
OTU1_Klebsormidium_flaccidum         ---------------------APESFKPDTSGLLTLLYR------PGHYDILYSL-----
OTU1_Haematococcus_pluvialis         ------------------------CAAAGAPIRVHVLYR------PGHYDILYKRPE---
OTU1_Chlamydomonas_reinhardtii       -------------------------CPPGTAPRVHLLYR------PGHYDILYAKSG---
OTU1_Gonium_pectorale                -------------------------CVPGTHPRVHLLYR------PGHYDILYAKGG---
OTU1_Volvox_carteri                  -------------------------CVPGTQPRVHLLYR------PGHYDILYPKGG---
OTU1_Chalmydomonas_eustigma          -------------------------GASVLRSCVSLLYR------PGHYDILYQQ-----
OTU1_Chromochloris_zofingiensis      ------------------------CTGADAVPHVHVLYR------PGHYDILYPL-----
OTU1_Raphidocelis_subcapitata        -------EGAGSIAAAAAGDGGGGAGTGRGRPSVHLLYR------PGHYDISYPKLIA--
OTU1_Scenedesmus_obliquus            --------------------LSAEEQAALGPPKVHMLYR------PGHYDILYPC-----
OTU1_Ulva_mutabilis                  ------------------------PVAGATIPQVAVLYR------PGHYDILYK------
OTU1_Caulerpa_lentillifera           -------------------------ASIKSEIIVHLLYR------PGHYDIIYPKTTSTL
OTU1_Cocomyxa_subellipsoidea         ----------------------LAESAAPVNPRVHVLYR------PGHYDILYP------
OTU1_Thalassiosira_pseudonana        ------------------------EEGSDEASTLTLLYR------PGHYDILYQSECRYI
OTU1_Phaeodactylum_tricornutum       -----------------------------ASIEISLLYR------PGHYDILYRCR----
OTU1_Phytophthora_sojae              -----------------------PATEQQEPVPITLLYR------PGHYDILYPREAGAE
OTU1_Trypanosoma_brucei              ----------------------SHDANEYTTIEVTLLYR------PGHYDLLYK------
OTU1_Laccaria_bicolor                --------------------------SGSESLPLVLLYR------PGHYDLLIKGKQGEQ
OTU1_Aspergillus_nigra               ---------------------------RPTGGTICLLYR------PGHYDLLYPAEPTVN
OTU1_Neurospora_crassa               ---------------------AISQPTSPLDPVICLLYR------PDHYDILYPVDPTPP
YOD1_Homo_sapiens                    -----------------------------YTKRVLLIYD------GIHYDPLQRNFPDPD
OTU1_Saccharomyces_cerevisiae        ------------------------------DNYILILFN------GIHYDSLTMNEFK--
OTUD6B_Homo_sapiens                  -------------------------------KPLILVYMRHAYGLGEHYNSVTRLVNIVT
OTU2_Saccharomyces_cerevisiae        ------------------------------NPELKLVYYKHSYALGEHYNSLHDS-----
OTUD5_Homo_sapiens                   -----------------------------EDEPIRVSYHR-----NIHYNSVVNPNKATI
OTUD3_Homo_sapiens                   ------------------------------VRELHIAYRY-----GEHYDSVRRINDNSE
```

b (8/8)

OTU2p_Chlamydomonas_reinhardtii	APGGQLVAAII------
OTU2m_Chlamydomonas_reinhardtii	APGGQLVAAII------
OTU2m_Chlamydomonas_incerta	APGGQLVAVDM------
OTU2m_Chlamydomonas_schloesseri	APGGQLVPAAKKGIVAT
OTU2m_Edaphochlamys_debaryana	APGGMLVPA--------
OTU2_Volvox_carteri	APGGALVPVLLPEGF--
OTU2_Gonium_pectorale	APGGALIPV--------
OTU2_Chromochloris_zofingiensis	STLTTLVKKAG------
OTU2_Raphidocelis_subcapitata	-----------------
OTU2_Ulva_mutabilis	CLVAQTGTVSDY-----
OTUB1_Homo_sapiens	-----------------
OTUB1_Mus_musculus	-----------------
OTUB_Drosophila_melanogaster	-----------------
OTUB_Caenorhapditis_elegans	SEIEN------------
OTUB_Nematostella_vectensis	-----------------
OTUB2_Homo_sapiens	-----------------
OTUB2_Mus_musculus	-----------------
OTUB_Monosiga_brevicolis	-----------------
OTU1_Arabidopsis_thaliana	SDNVGK-----------
OTU1_Medicago_tranculata	-----------------
OTU1_Oryza_sativa	-----------------
OTU1La_O.sativa_55840	-----------------
OTU1Lb_O.sativa_30974	NSSNPHEEKLLPIPSSI
OTU1Lc_O.sativa_32190	SGSKSSTQR--------
OTU1_Marchantia_polymorpha	APVPRVEEPQDL-----
OTU1_Physcomitrella_patens_181928	ALLSNLEQTG-------
OTU1_Klebsormidium_flaccidum	-----------------
OTU1_Haematococcus_pluvialis	-----------------
OTU1_Chlamydomonas_reinhardtii	-----------------
OTU1_Gonium_pectorale	-----------------
OTU1_Volvox_carteri	-----------------
OTU1_Chalmydomonas_eustigma	-----------------
OTU1_Chromochloris_zofingiensis	-----------------
OTU1_Raphidocelis_subcapitata	-----------------
OTU1_Scenedesmus_obliquus	-----------------
OTU1_Ulva_mutabilis	-----------------
OTU1_Caulerpa_lentillifera	-----------------
OTU1_Cocomyxa_subellipsoidea	-----------------
OTU1_Thalassiosira_pseudonana	R----------------
OTU1_Phaeodactylum_tricornutum	-----------------
OTU1_Phytophthora_sojae	QEKAS------------
OTU1_Trypanosoma_brucei	-----------------
OTU1_Laccaria_bicolor	ELRH-------------
OTU1_Aspergillus_nigra	MEPVVNYQYAMTSDYS-
OTU1_Neurospora_crassa	PAPMNIQVFRATIPQY-
YOD1_Homo_sapiens	TPPLTIFSSNDD-----
OTU1_Saccharomyces_cerevisiae	----TVFNKNQP-----
OTUD6B_Homo_sapiens	ENCS-------------
OTU2_Saccharomyces_cerevisiae	-----------------
OTUD5_Homo_sapiens	GVGLGLPSFKPG-----
OTUD3_Homo_sapiens	APAHLQTDFQML-----

a

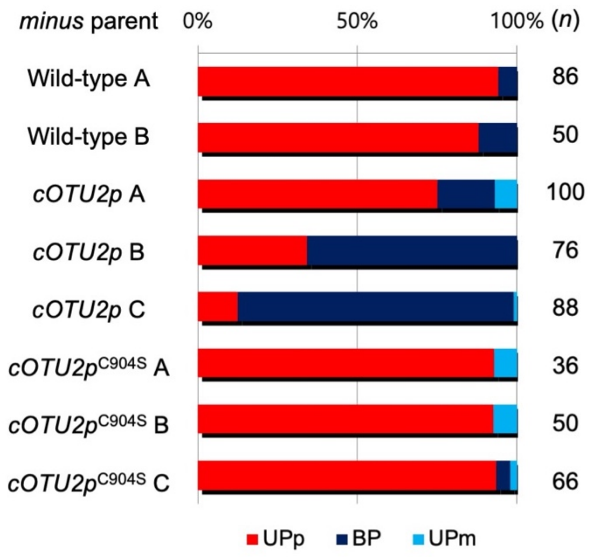

b

plus parent (eryR)	*minus* parent (specR)	Total (zygotes)	eryR	Both	specR	Exceptional	*P*-value
CC-333	Control-A	86	81	5	0	6%	n.a.
	Control-B	50	44	6	0	12%	n.a.
	cOTU2p-A	100	75	18	7	25%	7.77E-10
	cOTU2p-B*	76	26	50	0	66%	4.79E-49
	cOTU2p-C*	88	11	76	1	88%	9.66E-111
	cOTU2m-A	106	90	10	6	15%	5.12E-04
	cOTU2m-B	88	74	13	1	16%	3.31E-06
	cOTU2m-C	130	108	18	4	17%	2.64E-04
	cOTU2p^{C904S}-A	36	35	0	1	3%	8.26E-18
	cOTU2p^{C904S}-B	50	48	0	4	8%	5.88E-13
	cOTU2p^{C904S}-C	66	63	2	1	5%	1.47E-09

Supplementary Figure 10. DUB activity of Otu2p is required for promoting chloroplast DNA inheritance. Individual zygote progeny from the mating of the erythromycin-resistant (eryR) WT *plus* strain with three independent transgenic *minus* strains that harbor *cOTU2p* or *cOTU2p*^{C904S} (the non-catalytic Cys904Ser mutation) in a spectinomycin-resistant parental strain (specR) were examined for antibiotic resistance. (a) The C904S mutation abolished the increase in biparental inheritance caused by *cOTU2p*. (*n*) indicates the number of germinating zygotes examined. (b) Antibiotic resistance data depicted in Supplementary Fig. 10a. Asterisks indicate significant differences from the control mating in the exceptional zygote rate, as estimated with the chi-square test (*p*-value < 1.0E–25).

a

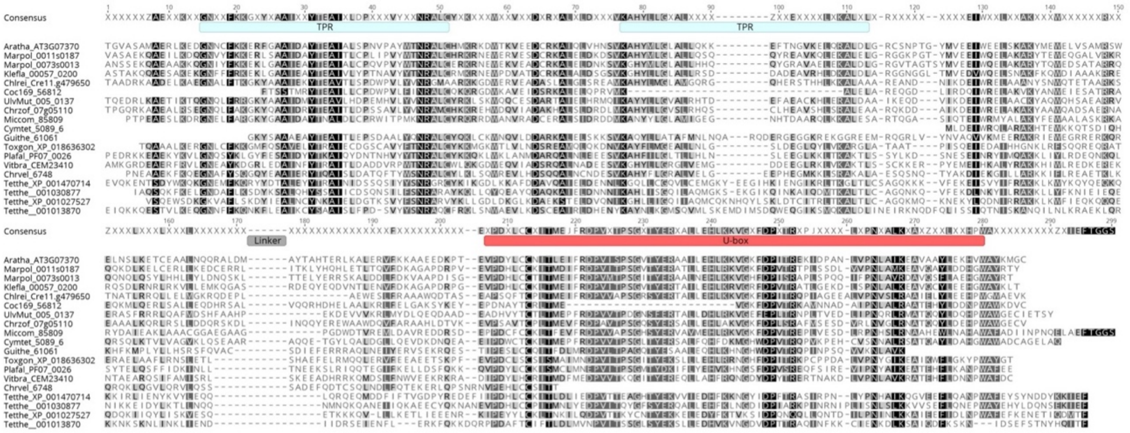

b

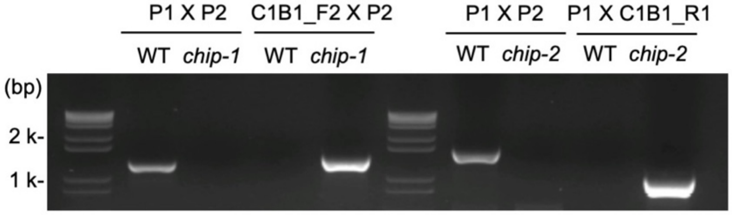

CLIP ID	Given name	P1 primer sequence	P2 primer sequence
LMJ.RY0402.080594	*chip-1*	CGAGATGAACAAGCACAGGA	TCAATTTGCTGCAGAACGTC
LMJ.RY0402.209732	*chip-2*	CAGGGAGTGTAGGAGCAAGC	CTATCTGCCTGGCCTACTGC
Insertion-specific	C1B1	F2-CGCTGATTGATACGGGATC	R1-TCGCTGAAAGTGGAGGTCAC

c

***chip-1* progeny**			UP/BP (UP%)		
	2-1-	3-2-	3-3-	CC-5325 *minus*	WT *minus*
1-2+	**100/33 (75.1%)**	**100/30 (76.9%)**	**100/28(78.1%)**	100/28(78.1%)	100/29 (77.5%)
1-4+	**100/34 (74.6%)**	**100/33 (75.1%)**	**100/31 (76.3%)**	100/31 (76.3%)	100/26 (79.3%)
2-2+	**100/28 (78.1%)**	**100/29 (77.5%)**	**100/26 (79.3%)**	100/27 (78.7%)	100/26 (79.3%)
WT *plus*	100/22 (82%)	100/24 (80.6%)	100/23 (81.3%)	100/21(82.6%)	100/20 (83.3%)
***chip-2* progeny**					
	1-2-	2-2-	4-2-	CC-5325 *minus*	WT *minus*
1-1+	**100/28 (78.1%)**	**100/29 (77.5%)**	**100/26(79.3%)**	100/30 (76.9%)	100/22 (82%)
2-1+	**100/26 (79.3%)**	**100/30 (76.9%)**	**100/25 (80%)**	100/33 (75.1%)	100/20 (83.3%)
3-1+	**100/24 (80.6%)**	**100/21 (82.6%)**	**100/24 (80.6%)**	100/26 (79.3%)	100/19 (84%)
WT *plus*	100/20 (83.3%)	100/24 (80.6%)	100/18 (84.7%)	100/18(84.7%)	100/16 (86.2%)

Supplementary Figure 11. CreCHIP is not required for selective degradation of cpDNA during zygote development. (a) CHIP E3 ligase contains two TPR repeats (blue bars) and a C-terminal U-box domain (red bar). Homologs were found in Viridiplantae, Cryptophytes, and Alveolates. (b) Two *chip* mutant strains were obtained from the CLiP collection *(44)*, with *aphVIII*-cassette insertions confirmed by CreCHIP-specific PCR using the P1-P2 primer pair and aphVIII-specific PCR using the F2 and R1 primers. The insertion-specific PCR was performed twice. (c) The percentage of uniparental zygotes in homozygous *CreCHIP* mutant zygotes was indistinguishable from that of wild-type (WT) zygotes. cpDNA in one chloroplast has degraded. Three *plus* and *minus CreCHIP* mutant progeny from a single backcross were examined by mating with WT *minus* and *plus* strains. Zygotes with cpDNA nucleoids in one and both chloroplasts were considered uniparental (UP) and biparental (BP), respectively. Counts stopped when the number of UP zygotes reached 100.

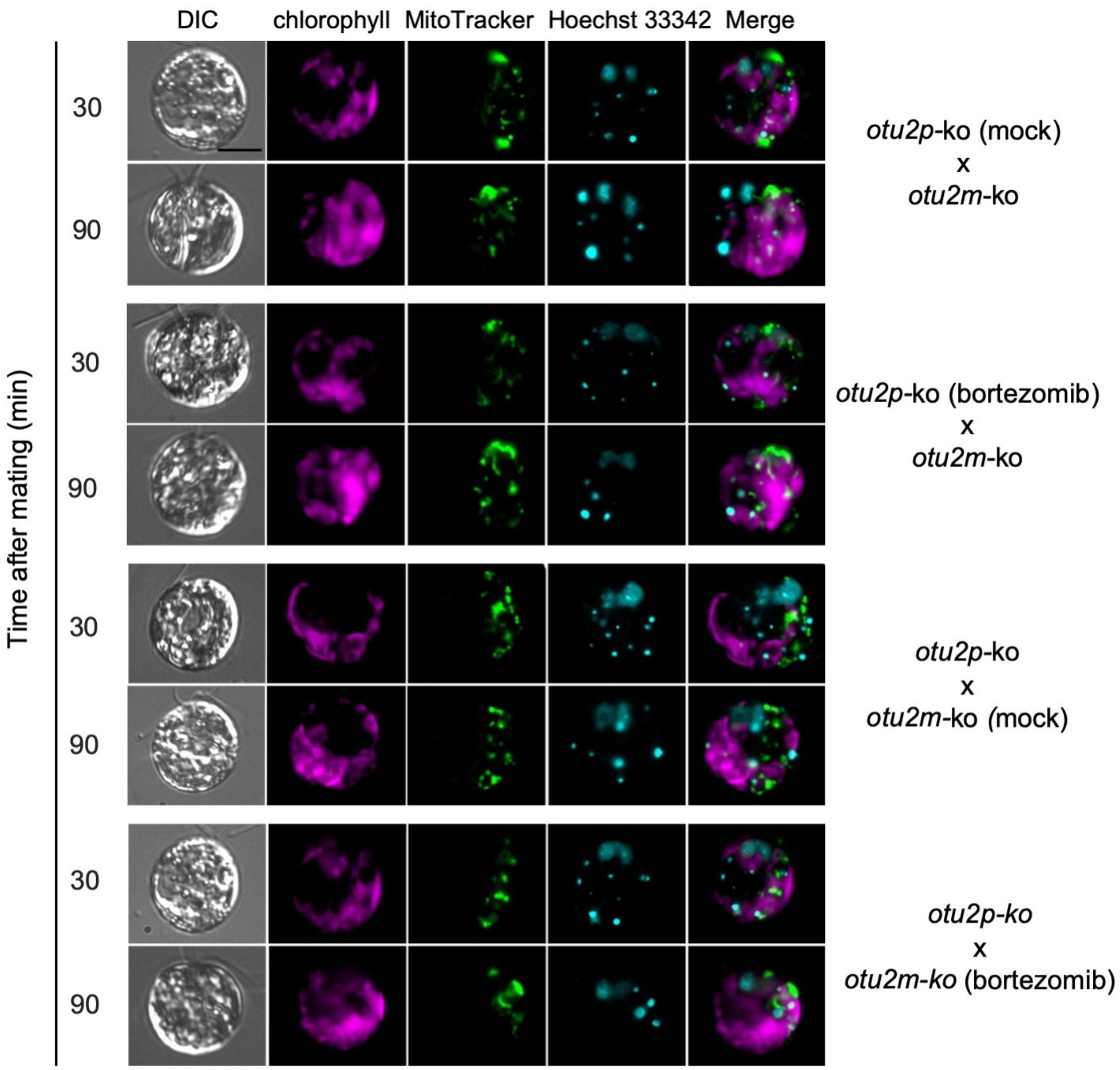

Supplementary Figure 12. Bortezomib treatment of *otu2*-ko mutant gametes phenocopied Otu2p expression for cpDNA degradation in zygotes. Differential interference contrast and overlaid fluorescent images of Hoechst 33342 (cyan)-stained zygotes with chlorophyll autofluorescence (magenta) at the indicated times after mating. Experiments were repeated three times. Quantitative results of three biological experiments are provided in Section "Figure 5d" of Supplementary Table 3. Only the *minus* gametes were stained with MitoTracker Green (green), staining *minus* chloroplasts in the zygotes. Bar = 5 µm.

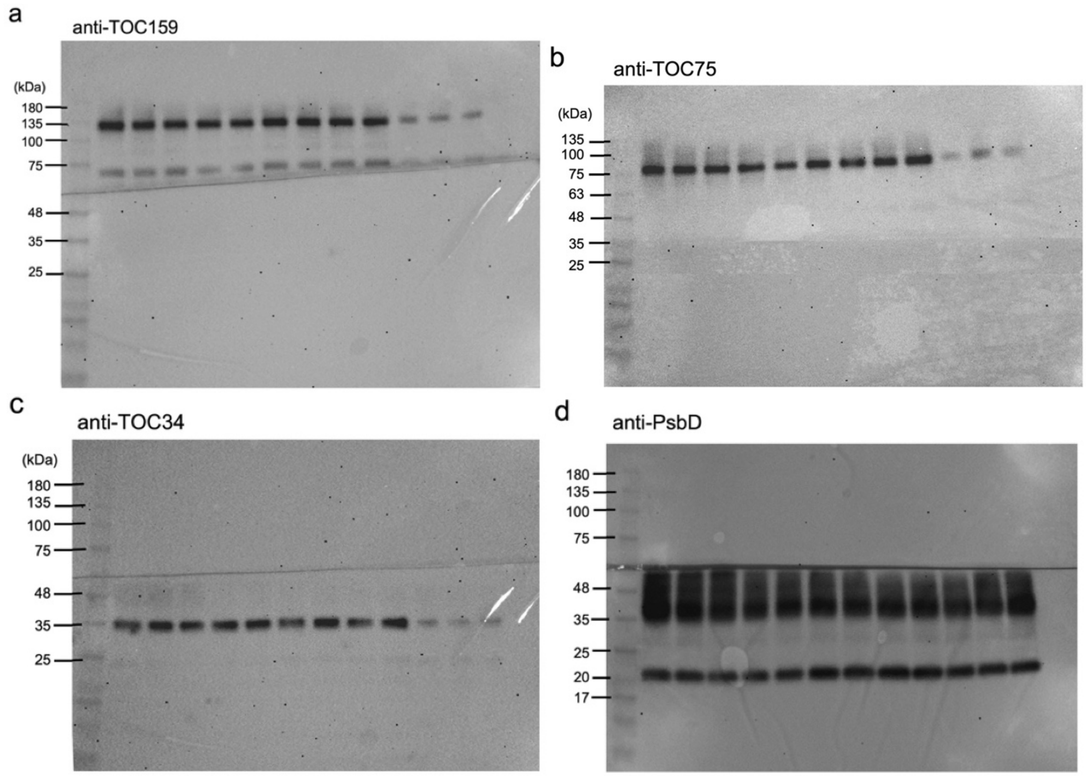

Supplementary Figure 13. The complete blots of Fig. 4a. (**a**) anti-TOC159. (**b**) anti-TOC75. (**c**) anti-TOC34. (**d**) anti-PsbD.

Supplementary Table 1. Genetic analysis of cpDNA inheritance for testing the "protector" function of *OTU2/EZY2* cluster in *MT+*

plus parent (specR)	minus parent (eryR)	Total (zygotes)	specR	Both	eryR	Exceptional	*P*-value
CJU10-J1	CC-2663	93	89	2	2	4%	n.a.
same as above	D13-03*	43	36	4	3	9%	2.06E-07
	D13-06	53	52	2	0	4%	1.22E-04
	D13-08*	73	43	30	0	28%	4.71E-39
	D13-10	84	84	0	0	0%	3.85E-02
	D13-13*	54	45	7	2	17%	1.21E-07
	D13-14*	66	49	11	6	26%	8.45E-15
	D13-19	94	87	4	3	7%	1.30E-01
	D13-20*	123	96	27	0	22%	1.00E-30
	D13-26	99	95	3	1	4%	5.25E-01
	D13-53	73	71	1	0	1%	1.90E-02
	D13-56	64	64	0	0	0%	9.00E-04
	D13-64	60	56	3	1	7%	4.69E-04
	D13-81	76	76	0	0	0%	1.52E-02
	P21-49	68	65	3	0	4%	9.52E-03
	O16-52	74	68	6	0	8%	1.47E-02

*Asterisks next to the strain name indicate significant difference from wild-type zygotes (CJU10-J1 x CC-2663) based on *chi*-square test (*p*-value <1.0E-6).

Supplementary Table 2. Genetic analysis of cpDNA inheritance for testing the "protector" function of the *OTU2p* and *OTU2m* coding segments

Strain ID	# zygote	UPp (eryR)	BP	UPm (specR)	(UPm + BP)	*P*-value
Control #1	40	32	2	6	20%	n.a.
AR::cOTU2p_#1	24	9	3	12	63%	1.94E-06
AR::cOTU2p_#7*	34	1	3	30	97%	2.48E-25
AR::cOTU2p_#8*	27	6	3	18	78%	8.03E-11
AR::cOTU2p_#9*	24	4	4	16	83%	7.07E-11
AR::cOTU2p_#10*	39	3	3	33	92%	7.31E-29
AR::cOTU2p_#11*	26	3	2	21	88%	1.63E-13
AR::cOTU2p_#15	98	79	7	12	19%	4.57E-20
AR::cOTU2p_#16	78	76	0	2	3%	7.49E-16
AR::cOTU2p_#17	31	22	1	8	29%	7.14E-02
AR::cOTU2p_#19	49	48	0	1	2%	1.71E-04
AR::cOTU2p_#20	61	51	3	7	16%	5.98E-04
AR::cOTU2p_#21	27	13	6	8	52%	7.11E-05
AR::cOTU2p_#22	29	13	11	5	55%	1.13E-05
AR::cOTU2p_#23*	39	13	13	13	67%	6.20E-13
AR::cOTU2p_#24	22	18	4	0	18%	4.37E-03

Strain ID	# zygote	UPp (eryR)	BP	UPm (specR)	(UPm + BP)	*P*-value
Control #1	83	72	8	3	13%	n.a.
Control #2	93	86	7	0	8%	n.a.
AR::cOTU2m_#2	96	92	2	2	4%	5.59E-10
AR::cOTU2m_#3	76	72	3	1	5%	3.05E-14
AR::cOTU2m_#4	98	85	3	10	13%	3.11E-09
AR::cOTU2m_#5	89	78	6	5	12%	4.87E-11
AR::cOTU2m_#6	96	89	1	6	7%	1.27E-09
AR::cOTU2m_#7	34	30	0	4	12%	9.70E-27
AR::cOTU2m_#8	92	86	5	1	7%	1.68E-10
AR::cOTU2m_#9	96	88	6	2	8%	1.47E-09
AR::cOTU2m_#10	41	39	2	0	5%	2.18E-24
AR::cOTU2m_#11	54	50	4	0	7%	3.45E-20
AR::cOTU2m_#12	81	72	4	5	11%	7.89E-13
AR::cOTU2m_#13	98	95	3	0	3%	8.59E-10
AR::cOTU2m_#14	78	70	6	2	10%	1.50E-13
AR::cOTU2m_#15	86	80	6	0	7%	9.13E-12
AR::cOTU2m_#16	104	90	12	2	13%	3.99E-08

(a) Asterisks indicate significant increase in exceptional zygote (UPm + BP) rates by chi-square test (*p*-value <1.0E-6). *AR::cOTU2p* #8, 10, and 23 and *AR::cOTU2m* #2, 6, and 12, showing comparable transgene expression with endogenous *OTU2,* were designated as A, B, and C, used in other experiments.

Supplementary Table 3. Summary of the detailed mating data described in this study

Cross		Mating efficiency	%[a]				Total QFC counted
plus	*minus*	%[a]	UP+ %	UP- %	BP %	No%	
**Figure 1**							
Haploid MT+	Haploid MT-	91.2 ± 1.3	85.0 ± 1.3	0.00	15.0 ± 1.3	0.0	608
Haploid MT+	MT+/MT-	88.5 ± 0.8	10.4 ± 4.1	1.3 ± 0.8	88.3 ± 4.8	0.0	1015
Haploid MT+	MT-/MT-	89.0 ± 1.6	78.5 ± 1.3	2.5 ± 0.5	19.0 ± 0.9	0.0	438
**Figure 2b**							
WT	cOTU2p	90.9 ± 1.1	12.2 ± 1.4	1.5 ± 0.8	86.3 ± 1.2	0.0	666
WT	cOTU2m	89.3 ± 0.8	81.1 ± 3.2	0.0	18.9 ± 3.2	0.0	416
**Figure 2c**							
WT	WT	92.5 ± 0.2	81.9 ± 1.8	0.2 ± 0.4	17.9 ± 2.2	0.0	543
otu2p	WT	89.2 ± 1.6	13.5 ± 2.8	1.1 ± 0.9	85.3 ± 2.1	0.0	770
otu2p	otu2m	87.2 ± 1.3	10.2 ± 1.2	3.6 ± 0.9	84.6 ± 0.6	1.6 ± 0.6	866
**Figure 2d**							
otu2p; cOTU2p	otu2m	89.4 ± 1.2	88.0 ± 0.2	1.2 ± 0.4	10.8 ± 0.4	0.0	866
otu2p	otu2m; cOTU2p	87.3 ± 0.4	2.6 ± 0.8	82.9 ± 3.3	14.6 ± 3.3	0.0	738
otu2p; cOTU2m	otu2m	87.6 ± 0.6	11.2 ± 0.3	0.8 ± 0.1	87.1 ± 0.4	0.9 ± 0.3	879
otu2p	otu2m; cOTU2m	88.5 ± 0.4	13.4 ± 0.7	1.3 ± 0.9	83.9 ± 1.6	1.3 ± 0.5	743
**Figure 5c**							
WT (mock)	WT (mock)	89.6 ± 2.2	82.4 ± 1.0	0.0	17.6 ± 1.0	0.0	1093
WT (MG132)	WT (mock)	88.8 ± 0.9	83.5 ± 1.3	0.0	16.5 ± 1.3	0.0	423
WT (bortezomib)	WT (mock)	88.1 ± 1.8	85.9 ± 1.8	0.0	83.9 ± 1.8	0.0	706
WT (mock)	WT (MG132)	88.6 ± 0.9	19.1 ± 1.7	0.0	80.9 ± 1.7	0.0	371
WT (mock)	WT (bortezomib)	87.2 ± 1.2	13.0 ± 0.7	0.0	87.0 ± 0.7	0.0	766
**Figure 5d**							
otu2p (mock)	otu2m	89.6 ± 0.9	15.5 ± 4.6	0.8 ± 0.4	83.7 ± 4.4	0.0	617
otu2p (MG132)	otu2m	88.2 ± 1.0	81.2 ± 0.4	1.8 ± 0.2	17.0 ± 0.4	0.0	441
otu2p	otu2m (mock)	89.6 ± 0.9	17.3 ± 1.7	4.1 ± 0.8	78.7 ± 1.7	0.0	435
otu2p	otu2m (MG132)	88.0 ± 0.5	1.0 ± 0.6	82.0 ± 1.8	17.1 ± 1.3	0.0	424
otu2p (bortezomib)	otu2m	86.1 ± 0.7	85.2 ± 0.8	1.2 ± 0.5	13.7 ± 0.5	0.00	689
otu2p	otu2m (bortezomib)	85.6 ± 1.5	0.8± 0.3	84.7 ± 1.0	14.6 ± 1.1	0.00	653

(a) % values show the average ± s.d. from three biological replicates.

Supplementary Table 4. Zygote-specific genes encoding chloroplast-localized proteins

Gene ID	FPKM (V-G-Z)[a]	Gene Name	Predicted locale[b]	(c)	(d)	Annotation
a. Putative DNA-binding proteins						
Cre06.g255750	0-0-1334	EZY1c	C	C	cTP	Localized to nucleoids
Cre06.g255650	0-1-411	EZY1b	C	C	cTP	Localized to nucleoids
Cre06.g255600	0-1-254	EZY1a	C	C	cTP	Localized to nucleoids
Cre02.g091550	46-33-868	EZY18/Whirly	CM	M	cTP	Localized to nucleoids
Cre07.g314650	41-36-408	EZY19/RECA	C	C	cTP	Localized to nucleoids
Cre12.g483720	0-0-127	OSB2/PTAC9	CM	C	mTP	Divergent
b. Topoisomerases						
Cre10.g442850	2-5-17	TOP-HMGbox, topo IA1	CM	C	mTP	
Cre04.g221650	1-1-13	Topo IV (GYRA)	C	C	cTP	
Cre10.g440750	4-1-45	Topo IV (GYRB)	O	O	mTP	
c. Candidate helicases						
Cre06.g296150	3-3-23	PIF1-type helicase	CM	C	mTP	PIF1 domain only
Cre06.g256700	1-1-38	AAA_helicase	C	C	cTP	Divergent
Cre06.g256650	0-0-13	AAA_helicase	M	M	mTP	Divergent
d. DNA replication enzymes						
Cre17.g736150	1-0-14	DNA polymerase	M	M	mTP	Divergent

(a) Annotation and RNA-seq-based expression measures (FPKM) are taken from Joo et al. (*4*).
(b) Predicted organellar localization is indicated as C (Chloroplast), M (Mitochondria), or O (others).
(c) Prediction by TargetP (https://services.healthtech.dtu.dk/service.php?TargetP-2.0).
(d) Prediction by PredAlgo (http://lobosphaera.ibpc.fr/cgi-bin/predalgodb2.perl?page=main).

Supplementary Table 5. Genetic linkage of OTU2 homologs to the mating-type loci in Volvocine algae

Genome	*OTU2*	MT-linkage	mating-type (location)	*MTD1*	*SAD1*
C. reinhardtii[a]	*MT+/MT-*	Yes	*MT+* (Ch6)	*MT-*	*MT+/MT-*
C. incerta[b]	C0033	Yes	*MT-* (C0033)	C0033	C0033
C. schloesseri[b]	C0045	Yes	*MT-* (C0045)	C0045	C0045
Edaphochlamys debaryana[b]	C0043	Yes	*MT-* (C0043)	C0043	C0043
Gonium pectorale[c]	C000113	No	*MT-* (C000001)	C000001	C000143
Volvox carteri[d]	Sc35	No	*MT+* (Sc2, Sc38, Sc80)	Pseudogene	Sc27
Dunaliella salina[e]	C0044	Unknown	*MT-* (n.d.)	C0036	C0945

a) De Hoff et al. (2013) (*5*); b) Craig et al. (2021) (*6*); c) Hamaji et al. (2016) (*7*); d) Ferris et al. (2010) (*8*); e) Polle et al. (2017) (*9*).

Supplementary Table 6. List of primers used in this study

Name	Sequence	Restriction enzyme tag
psbB_qF1	AGCTGTTTTACCTGTACGTGG	
psbB_qR1	TCTGGAGTTATGAAGGTGTTGC	
psbD_qF1	GCTAACAGTATGGCTCACTCTC	
psbD_qR1	AAGCAACGAATGCCCATAAAC	
Cblp_qF1	ACATTTCCTGACCTCCACATC	
Cblp_qR1	TGCTGGTGATGTTGAACTCG	
RCK1_qF1	GCCACACCGAGTGGGTGTCGTGCG	
RCK1_qR1	CCTTGCCGCCCGAGGCGCACAGCG	
OTU2_F1	ACGGCAACTGCTTCTACC	
OTU2_F2	CTTGGCAATCAGGAGGCAG	
OTU2_F3	CATGACCGGGTTGACGGAC	
OTU2_F4	GCACTCGAGATCTGAGCAG	
OTU2_F5	GGTAGTACGGTTCGGCTAC	
OTU2_F6	CATGCATGCTTCGCCGG	
OTU2_F7	CTACAGTACCCTTGCTGGCAG	
OTU2_F9	TACCCGTGGCAACGCGAGTAC	
OTU2_F11	GAGGCGCATCAACTGGAATCG	
OTU2_F12	CTGGACTGTGACAAGCTCATC	
OTU2_F13	CTAGGACGGACCGACTTGGTC	
OTU2_F14	GCAGTCATGGTAGTCACCTTG	
OTU2_F16	ATGGCGCGCAAGGTCCGCGAGA	
OTU2_F17	CAGCGGCTGCTACGCCTTTTG	
OTU2_F18	CATATGACCGGGTTGACGGAC	NdeI
OTU2_F21	AGCAACGGCGCCGAGCCG	
OTU2p_R1	CCGGCGAAGCATGCATG	
OTU2m_R1	CTCTGCTCGGCGCTGTTG	
OTU2_R2	GGTAGAAGCAGTTGCCGTC	
OTU2_R3	CAGCTGCTCAACCTCCAC	
OTU2_R4	GCTGCTCAGATCTCGAGTG	
OTU2_R5	CGTAGCCGAACCGTACTAC	
OTU2_R12stop	gaattcTTATATAATTGCCGCCACTAG	EcoRI
OTU2_R12nostop	gaattcTATAATTGCCGCCACTAGTTGC	EcoRI
OTU2_R14	CAGCACACTGGCTTGCACAG	
OTU2_R16	AGGATCCCTGCAGGCGCTGCATCTC	BamHI
OTU2_R20	TGATGATGCCGGGGCTGC	
OTU2_R21	GTGCGAAGCGGATCACCTC	
OTU2_C904S_F	GGGGCGACGGCAACAGCTTCTACCGCGCG	
OTU2_C904S_R	CGCGCGGTAGAAGCTGTTGCCGTCGCCCC	
rbcsT_R1	AGCGCCTCCATTTACAC	
AtOTU1_F1	catATACGAACGCGTGCCGATTT	NdeI
AtOTU1_R1	gaattcTCATTTCCCCACATTGTCTG	EcoRI
OTU2_ko_F1	CGCGTCGGCCGCCGCCTCTG	
OTU2_ko_R1p	GCAGCCGTACTTGGCGCAGTG	
OTU2_ko_R1m	GCAGCCGTACTCGACGCAGTG	

Supplementary References 1–9

1. Kuroiwa, T., Kawano, S., Nishibayashi, S. & Sato, C. Epifluorescent microscopic evidence for maternal inheritance of chloroplast DNA. *Nature* **298**, 481-483 (1982).

2. Matagne, R. F. & Mathieu, D. Transmission of chloroplast genes in triploid and tetraploid zygospores of Chlamydomonas reinhardtii: roles of mating-type gene dosage and gametic chloroplast DNA content. *Proc. Natl. Acad. Sci. U. S. A.* **80**, 4780-4783 (1983).

3. Ferris, P. J., Armbrust, E. V. & Goodenough, U. W. Genetic structure of the mating-type locus of Chlamydomonas reinhardtii. *Genetics* **160**, 181-200 (2002).

4. Joo, S. et al. Gene regulatory networks for the haploid-to-diploid transition of Chlamydomonas reinhardtii. *Plant Physiol.* **175**, 314-332 (2017).

5. De Hoff, P. L. et al. Species and population level molecular profiling reveals cryptic recombination and emergent asymmetry in the dimorphic mating locus of C. reinhardtii. *PLoS Genet.* **9**, e1003724. https://doi.org/10.1371/journal.pgen.1003724 (2013).

6. Craig, R. J., Hasan, A. R., Ness, R. W. & Keightley, P. D. Comparative genomics of Chlamydomonas. *Plant Cell* **33**, 1016-1041 (2021).

7. Hamaji, T. et al. Sequence of the Gonium pectorale Mating Locus reveals a complex and dynamic history of changes in Volvocine algal mating haplotypes. *G3 Genes Genomes Genet.* **6**, 1179–1189 (2016).

8. Ferris, P. et al. Evolution of an Expanded Sex-Determining Locus in Volvox. *Science* **328**, 351–354 (2010).

9. Polle, J. E. W. *et al.* Draft nuclear genome sequence of the halophilic and beta-carotene-accumulating green alga Dunaliella salina Strain CCAP19/18. *Genome Announc.* 5:e01105-17. https://doi.org/10.1128/genomeA.01105-17 (2017).
