# Supplementary material for: Sex-linked deubiquitinase establishes uniparental transmission of chloroplast DNA
Source: Nat Commun. 2022 Mar 3;13:1133. doi: 10.1038/s41467-022-28807-6 (PMC8894339; doi:10.1038/s41467-022-28807-6)
Supplement: Supplementary file 2 — Reporting Summary [file 41467_2022_28807_MOESM2_ESM.pdf]

nature portfolio

Corresponding author(s): Jae-Hyeok Lee

Last updated by author(s): Jan 25, 2022

Reporting Summary

Nature Portfolio wishes to improve the reproducibility of the work that we publish. This form provides structure for consistency and transparency in reporting. For further information on Nature Portfolio policies, see our Editorial Policies and the Editorial Policy Checklist.

Statistics

For all statistical analyses, confirm that the following items are present in the figure legend, table legend, main text, or Methods section.

n/a	Confirmed	
☐	☒	The exact sample size (n) for each experimental group/condition, given as a discrete number and unit of measurement
☐	☒	A statement on whether measurements were taken from distinct samples or whether the same sample was measured repeatedly
☐	☒	The statistical test(s) used AND whether they are one- or two-sided<br>*Only common tests should be described solely by name; describe more complex techniques in the Methods section.*
☒	☐	A description of all covariates tested
☒	☐	A description of any assumptions or corrections, such as tests of normality and adjustment for multiple comparisons
☐	☒	A full description of the statistical parameters including central tendency (e.g. means) or other basic estimates (e.g. regression coefficient) AND variation (e.g. standard deviation) or associated estimates of uncertainty (e.g. confidence intervals)
☐	☒	For null hypothesis testing, the test statistic (e.g. F, t, r) with confidence intervals, effect sizes, degrees of freedom and P value noted<br>*Give P values as exact values whenever suitable.*
☐	☒	For Bayesian analysis, information on the choice of priors and Markov chain Monte Carlo settings
☒	☐	For hierarchical and complex designs, identification of the appropriate level for tests and full reporting of outcomes
☒	☐	Estimates of effect sizes (e.g. Cohen's d, Pearson's r), indicating how they were calculated

Our web collection on statistics for biologists contains articles on many of the points above.

Software and code

Policy information about availability of computer code

Data collection	HMMsearch (v3.1b2) was used to collect OTUB family proteins sequences.
Data analysis	Image Lab software 6.1 from Biorad was used to quantify bands in immunoblots.<br>MAFFT algorithm was used to align protein sequences.<br>IQ-TREE software was used to generate the maximum-likelihood phylogeny of OTU domain.<br>CAS-designer was used to design CAS9-guide RNAs.

For manuscripts utilizing custom algorithms or software that are central to the research but not yet described in published literature, software must be made available to editors and reviewers. We strongly encourage code deposition in a community repository (e.g. GitHub). See the Nature Portfolio guidelines for submitting code & software for further information.

Data

Policy information about availability of data

All manuscripts must include a data availability statement. This statement should provide the following information, where applicable:

- Accession codes, unique identifiers, or web links for publicly available datasets
- A description of any restrictions on data availability
- For clinical datasets or third party data, please ensure that the statement adheres to our policy

The Chlamydomonas lines generated in this study are deposited and available at the Chlamydomonas Resource Center (https://www.chlamycollection.org) with the following accession numbers.
CC-5776 cOTU2p mt-; CC-5778 cOTU2m mt-; CC-5779 otu2p-ko mt+ ; CC-5780 otu2m-ko mt-; CC-5781 chip-1 mt-; CC-5782 chip-2 mt-
The sequences of OTU2p-A and OTU2m clones are deposited to GenBank with accession numbers OL770090 for OTU2p-A and OL770091 for OTU2m.

Field-specific reporting

Please select the one below that is the best fit for your research. If you are not sure, read the appropriate sections before making your selection.

☒ Life sciences ☐ Behavioural & social sciences ☐ Ecological, evolutionary & environmental sciences

For a reference copy of the document with all sections, see nature.com/documents/nr-reporting-summary-flat.pdf

Life sciences study design

All studies must disclose on these points even when the disclosure is negative.

Sample size	Sample sizes of the microscopic data were determined by the criterion that at least 100 cells are collected per category to ensure statistical analysis and reproduction of the experiments.
Data exclusions	No data were excluded from this study.
Replication	Experiments were performed at least twice with similar results.
Randomization	Randomization is not relevant because all samples were grown in defined media, and were prepared and harvested from plates maintained in the controlled environment.
Blinding	Blinding is not relevant because each experiment was performed by single researcher. Microscopic Chlamydomonas cells were analyzed using objective, standardized protocols that include appropriate controls.

Reporting for specific materials, systems and methods

We require information from authors about some types of materials, experimental systems and methods used in many studies. Here, indicate whether each material, system or method listed is relevant to your study. If you are not sure if a list item applies to your research, read the appropriate section before selecting a response.

Materials & experimental systems

n/a	Involved in the study	
☐	☒	Antibodies
☐	☒	Eukaryotic cell lines
☒	☐	Palaeontology and archaeology
☒	☐	Animals and other organisms
☒	☐	Human research participants
☒	☐	Clinical data
☒	☐	Dual use research of concern

Methods

n/a	Involved in the study	
☒	☐	ChIP-seq
☒	☐	Flow cytometry
☒	☐	MRI-based neuroimaging

Antibodies

Antibodies used	anti-TOC34 Arabidopsis (Agrisera AS07-238); anti-TOC75 pea (gift from Enrico Shleiff, detecting a major product at 75 kDa and non-specific product at 35–48 kDa); anti-TOC159 pea (gift from Bettina Bolter, detecting a major product just below 180 kDa and minor product at 75–100 kDa); anti-PsbD pea (Agrisera AS06-146)
Validation	Specificity of the anti-TOC34 antibody has been confirmed by the manufacturer and published on the website (https://https://www.agrisera.com/en/artiklar/toc34-arabidopsis-thliana.html).<br>Specificity of the anti-TOC75 antibody has been published in Schottkowski et al. (Proc. Natl. Acad. Sci. USA 109:19286-19291, 2012).<br>Specificity of the anti-TOC159 antibody has been published in Formighieri et al. (Plant J. 73:850-861, 2013).<br>Specificity of the anti-PsbD antibody has been confirmed by the manufacturer and published on the website (https://www.agrisera.com/en/artiklar/psbd-d2-global-antibody.html).

Eukaryotic cell lines

Policy information about cell lines

Cell line source(s)	All the Chlamydomonas strains used in this study were obtained from the Chlamydomonas Resource Center.
Authentication	Wild-type strains received from the Chlamydomonas Resource Center were validated by their gametic phenotype or antibiotic resistance. Mutant alleles were validated by southern blotting or PCR using genomic DNAs.

Mycoplasma contamination	*Confirm that all cell lines tested negative for mycoplasma contamination OR describe the results of the testing for mycoplasma contamination OR declare that the cell lines were not tested for mycoplasma contamination.*
Commonly misidentified lines<br>(See ICLAC register)	*Name any commonly misidentified cell lines used in the study and provide a rationale for their use.*
